# Timing of adverse childhood experiences shapes epigenetic ageing and life-history outcomes

**DOI:** 10.1038/s41598-026-58459-1

**Published:** 2026-06-19

**Authors:** Wesley J. Wang, Evelina T. Akimova

**Affiliations:** 1 Department of Sociology, Purdue University, West Lafayette, IN USA; 2 Center on Aging and the Life Course, Purdue University, West Lafayette, IN USA; 3 Department of Statistics, Purdue University, West Lafayette, IN USA; 4 The Methodology Center at Purdue (MCAP), Purdue University, West Lafayette, IN USA; 5 Interdisciplinary Genomics Research Center (IGRC), Purdue University, West Lafayette, IN USA; 6 Present Address: Department of Sociology, Princeton University, Princeton, NJ USA

**Keywords:** Developmental biology, Health care, Psychology, Psychology, Risk factors

## Abstract

Early-life adversity is widely linked to accelerated biological ageing, yet it remains unclear whether such associations reflect exposure during sensitive developmental periods, the cumulative burden of exposures, or temporal proximity to later outcomes. Here, we leverage life-history theory and a life course framework to nuance how the timing of adverse childhood experiences (ACEs) becomes biologically embedded through epigenetic ageing. Using longitudinal data from the Future of Families and Child Wellbeing Study (N=1,974), we apply statistical learning and structured life course modelling to test sensitive period, cumulative risk, and recency hypotheses across multiple domains of adversity (poverty, instability, deprivation, and maltreatment). We find that adversity exposure during specific developmental periods, rather than cumulative burden or recent exposure, are most strongly associated with epigenetic age acceleration in late childhood ($R^2_{pooled\ ACEs}$=0.003). Moreover, the timing and direction of these effects vary by adversity type. Epigenetic ageing is in turn associated with later health-related risks ($\beta _{bmi}$=0.29, SE=0.06; $OR_{depression}$=1.62, SE=0.27) and demographic behaviour ($\beta _{no.\ births}$=0.21, SE=0.08; $\beta _{no.\ pregnancies}$=0.22, SE=0.11), and further mediates the association between ACEs and outcomes in young adulthood, particularly for BMI ($\beta _{bmi}$=0.003, SE=0.002, *prop.\ mediated*=11%). These findings demonstrate that childhood adversity may be linked to biological ageing in developmentally specific and domain-dependent ways, with certain developmental periods appearing more sensitive to adversity exposure than others.

## Background

Childhood adversity is a well-established predictor of later-life health, morbidity, and reproductive outcomes^1,2^. Individuals exposed to adverse childhood experiences (ACEs) face elevated risks of chronic disease, mental illness, and earlier reproductive transitions across diverse social contexts. Despite extensive evidence documenting these associations, the life course processes through which early adversity becomes biologically embedded remain poorly understood. In particular, it is unclear whether adversity exerts its influence primarily through cumulative exposure across childhood, through recent experiences proximal to later outcomes, or through increased sensitivity during specific developmental periods.

Life-history theory provides a powerful framework for understanding how childhood adversity shape long-term developmental trajectories. From an evolutionary perspective, life-history theory conceptualises development as a process of adaptive allocation of limited energetic resources across competing demands of physical growth, somatic maintenance, and reproduction^3^. Early life environments calibrate these allocations, such that exposure to harsh or unpredictable conditions prioritises earlier reproduction at the expense of long-term health and longevity^4^. Conversely, stable and resource-rich environments permit slower maturation, delayed reproduction, and greater investment in long-term health^5^.

In recent years, life-history theorists have increasingly emphasised that qualitatively distinct environmental conditions (e.g., unpredictability, harshness, and threat) experienced by children may exert differentiated developmental effects^6–10^. Related frameworks, such as the psychosocial acceleration theory^10^ and life course perspectives^11^, further propose that developmentally sensitive periods may shape how adversity influences reproductive and behavioural outcomes, especially during early childhood^7,10^. However, many empirical studies examining the effects of childhood adversity and epigenetic ageing rely on cumulative exposure indices or ever-exposed measures to quantify adversity experienced by children^12–14^. While such measures capture overall burden, they provide comparatively less insight into whether adversity experienced at different developmental stages translate into distinct effects on epigenetic ageing.

Disentangling these life course processes is critical for understanding how early adversity shapes later-life outcomes. If biological ageing reflects cumulative wear and tear, then prolonged exposure should be most predictive of future outcomes. If recency dominates, then more proximal stressors should exert stronger effects. Alternatively, if sensitive periods govern biological embedding, then adversity encountered during specific developmental windows may have disproportionate and lasting consequences, even when total exposure is limited. These competing hypotheses imply fundamentally different mechanisms linking childhood environments to adult health and reproductive trajectories.

Epigenetic ageing provides a measurable biological pathway through which these processes may operate. Epigenetic clocks capture variation in biological ageing relative to chronological age and are robustly associated with morbidity, mortality, and functional decline^15,16^. Accelerated epigenetic ageing has been linked to a range of adverse social and environmental exposures, including childhood adversity^17–19^. However, most existing studies rely on cumulative measures of adversity (e.g.^20–22^), leaving unresolved whether epigenetic age acceleration reflects early developmental calibration, ongoing exposure, or more recent stressors.

**Fig. 1:**
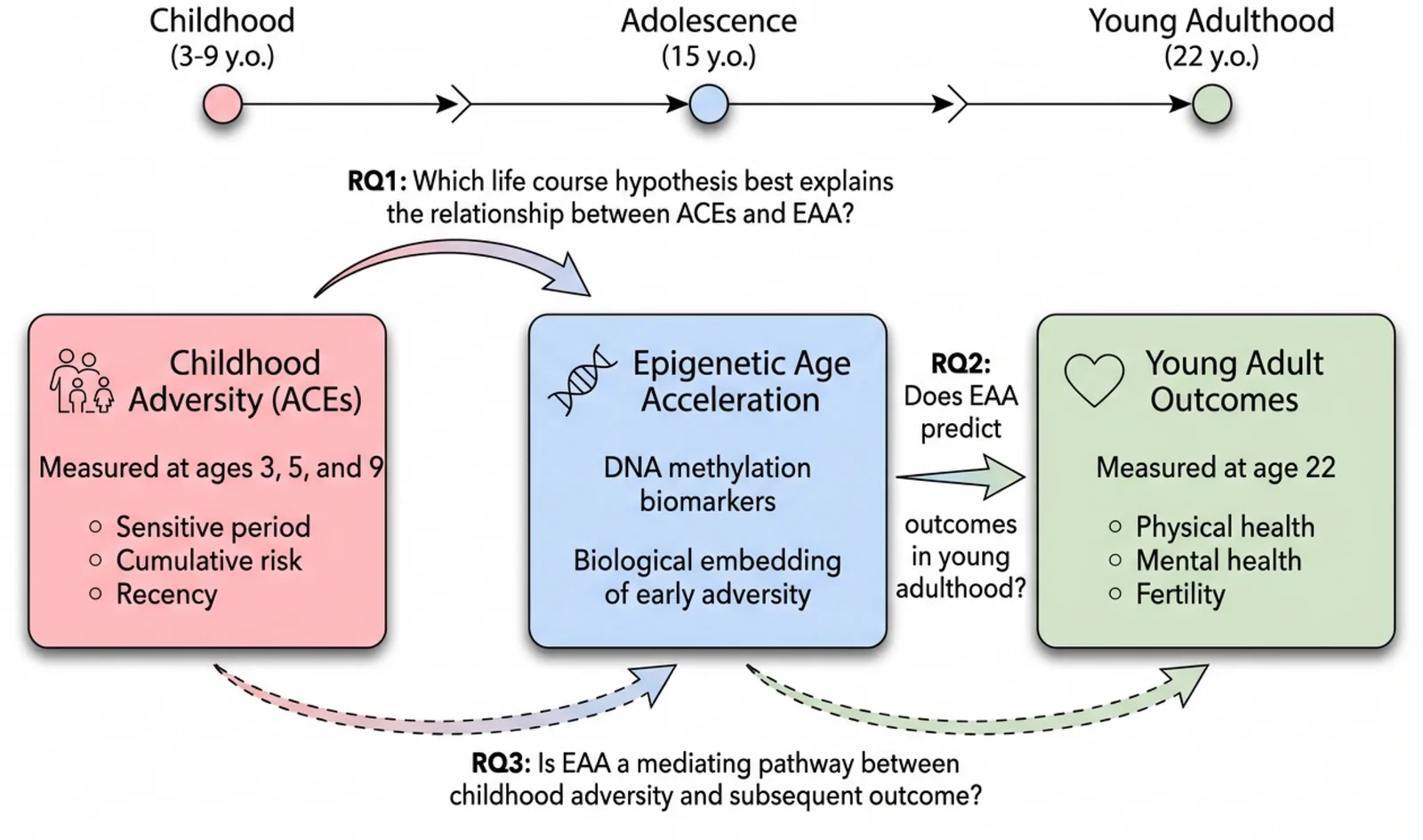
Overview of the Study.

In this study, we integrate life-history theory with a life course framework to examine how childhood adversity shapes epigenetic ageing and downstream outcomes. Using longitudinal data from the Future of Families and Child Wellbeing Study, we explicitly test three competing life course hypotheses—sensitive periods, cumulative risk, and recency—across multiple domains of adversity experienced between ages 3 and 9. We then examine whether epigenetic ageing predicts anthropometric, mental health, and reproductive outcomes in young adulthood, and whether it mediates the association between childhood adversity and these outcomes (see Fig. 1 for the study overview).

By explicitly addressing competing life course models, this study advances our understanding of how early-life environments become biologically embedded. Rather than treating childhood adversity as a uniform or cumulative exposure, we show that the timing and domain of adversity matter for biological ageing trajectories. In doing so, we refine the application of life-history theory to human development and identify sensitive developmental windows through which social environments may contribute to long-term biological and life-history outcomes.

## Results

### Key life course hypotheses of childhood adversity

Epigenetic ageing was most strongly predicted by the *timing* of childhood adversities within specific developmental windows, rather than by their cumulative or recent occurrence. Using the Structured Life Course Modelling Approach (SLCMA) with least-angle regression, we compared three competing life course hypotheses—sensitive periods, recency, and cumulative risk—to determine which best accounted for variation in epigenetic age acceleration (see Methods). The model contributing the greatest improvement $R^2$ was retained as the most salient representation of how childhood adversity shapes biological ageing. Results are shown in Table 1 below.

Among the three life course hypotheses tested, *sensitive periods* explained the largest share of variance in epigenetic age acceleration (improvement $R^2$ = 0.0022 – 0.0030). The magnitude of these improvements in $R^2$ is consistent with prior applications of SLCMA to epigenetic ageing outcomes, including those reported by Marini et al. (2020)^23^.

An age 9 sensitive period emerged as most salient for pooled adverse childhood experiences (improvement $R^2$ = 0.0027, *p <0.05*) and for poverty (improvement $R^2$ = 0.0028, *p <0.05*), whereas age 3 was the critical window for familial instability (improvement $R^2$ = 0.0030, *p <0.05*) and deprivation (improvement $R^2$ = 0.0022, *p <0.05*). For maltreatment-related exposures, sensitive periods at ages 3 (improvement $R^2$ = 0.0002) and 9 (improvement $R^2$ = 0.0009) were detected, but did not reach statistical significance even after sequential removal of non-significant predictors (*p >0.05*). Results were robust to alternative operationalizations of life course hypotheses (see *Sensitivity Analyses* subsection of the Methods section, and Tables S5–S6 of the Supplementary Information).

**Table 1:** Selected Life Course Models.

Adversity	DunedinPACE clock		
	Model selected	p-value	Improvement R$^2$
Pooled ACEs	Sensitive period (Age 9)	**0.032**	**0.003**
Poverty	Sensitive period (Age 9)	**0.025**	**0.003**
Instability	Sensitive period (Age 3)	**0.011**	**0.003**
	Sensitive period (Age 9)	0.428	0.004
Deprivation	Sensitive period (Age 3)	**0.022**	**0.002**
Maltreatment	Sensitive period (Age 3)	0.413	< 0.001
	Sensitive period (Age 9)	0.529	< 0.001

Bold values indicate statistical significance at *p<0.05*. Incremental R$^2$ represents the increase in variance explained by the selected life course model relative to null model

### Sensitive period effects of ACEs on biological ageing

To complement the identification of sensitive developmental periods, multivariable OLS regressions were performed to assess the significance and magnitude of associations between children’s sensitive ages and their subsequent pace of biological ageing across adversity domains (Table 2). Adversity experienced at sensitive age 9 was associated with an *accelerated* pace of ageing for Pooled (*β =0.0108*; *se =0.0046*; *p<0.05*) and Poverty-related ACEs (*β =0.0207*; *se =0.0088*; *p<0.05*). In contrast, adversity at sensitive age 3 was associated with a *decelerated* pace of ageing for Instability (*β =-0.0266*; *se =0.0101*; *p<0.01*) and Deprivation (*β =-0.0317*; *se=0.0140*; *p<0.05*). Although sensitive periods at ages 3 (*β =0.0110*, *se =0.0116*, *p >0.05*) and 9 (*β =0.0086*, *se =0.0112*, *p >0.05*) were identified for Maltreatment, none of these life course models significantly predicted subsequent epigenetic ageing.

Prior research has also demonstrated the salient influence of children’s socioeconomic conditions, particularly at birth, on subsequent epigenetic outcomes. For instance, childhood SES at birth was found to be most strongly predictive of epigenetic indices of BMI at age 9 within the FFCWS cohort^24^, suggesting that epigenetic signatures may reflect socioeconomic environments into which children are born. We, therefore, also tested for children’s poverty status at birth as sensitivity analyses, to examine if socioeconomic conditions at birth also constituted a sensitive period. Results suggest that poverty at age 9 still remained the primary sensitive period for later life epigenetic ageing. Although our analysis identified both poverty at birth and age 9 as sensitive periods (Supplementary Figure S22), multivariate OLS results indicate that only age 9 remained predictive of subsequent epigenetic ageing after sequential removal of non-significant predictors (Supplementary Table S7), suggesting that SES at birth is not strongly related to subsequent pace of ageing.

We additionally estimated models adjusted for saliva cell-type composition. Because DunedinPACE was originally developed using whole-blood methylation data, estimated saliva cell composition may partly capture tissue heterogeneity relevant to cross-tissue measurement comparability^25^. At the same time, immune-related variation in saliva composition may itself reflect biological embedding processes linked to childhood adversity and chronic inflammation^26,27^. We therefore present models adjusted for estimated saliva epithelial and immune cell composition to assess robustness to saliva tissue composition, while interpreting earlier unadjusted models as estimating the total association between childhood adversity and DunedinPACE ageing, including immune- and inflammation-related processes reflected in saliva cell composition.

After adjusting for estimated epithelial cell composition, only the age 9 sensitive period remained significant in the pooled ACEs model (*β =0.007*, *se =0.004*, *p<0.05*). Previously identified sensitive periods for *Poverty* (*β =0.012*, *se =0.007*, *p>0.05*), *Instability* (*β =-0.015*, *se =0.008*, *p>0.05*), and *Deprivation* (*β =0.014*, *se =0.011*, *p>0.05*) attenuated in significance (Supplementary Table S8). This attenuation suggests that observed associations may partly reflect immune-related biological pathways and/or saliva tissue heterogeneity relevant to methylation measurement. However, estimated saliva cell composition may not simply represent nuisance variation to be controlled. In this context, adjustment for cell composition may partially account for downstream biological processes through which adversity becomes biologically embedded.

To further examine this possibility, we conducted mediation analyses assessing whether estimated epithelial cell composition statistically mediated associations between childhood adversity and DunedinPACE (Supplementary Table S11). Mediation analyses indicated substantial mediation for *Instability* (*β =-0.020*; *prop.mediated = 76%*; *p <0.05*) and *Deprivation* (*β =-0.025*; *prop.mediated = 85%*; *p <0.05*), but not for *Pooled ACEs* (*β =0.002*; *prop.mediated = 18%*; *p >0.05*) or *Poverty* (*β =0.003*; *prop.mediated = 17%*; *p >0.05*). These findings suggest that attenuation following cell-composition adjustment likely captures the removal of biologically meaningful downstream processes linked to adversity exposure.

**Table 2:** Comparison of Life Course Models Linking Childhood Adversity to Epigenetic Age Acceleration in Late Childhood (Final Model).

	DunedinPACE Ageing				
	Pooled ACEs	Poverty	Instability	Deprivation	Maltreatment
Life Course Models					
Sensitive Period (Age 3)			**-0.0266**$$^{**}$$	**-0.0317**$$^{*}$$	0.0110
			(0.0101)	(0.0140)	(0.0116)
Sensitive Period (Age 9)	**0.0108**$^{*}$	**0.0207**$^{*}$			0.0086
	(0.0046)	(0.0088)			(0.0112)
Observations	1,974	1,974	1,974	1,974	1,974
R$^2$	0.05960	0.05958	0.06023	0.05938	0.05781
Adjusted R$^2$	0.05673	0.05671	0.05736	0.05651	0.05446

* p<0.05, ** p<0.01, *** p<0.001 Models adjust for sex, race, maternal race, maternal education, and maternal age at birth

### Predictive role of EAA in young adult health and life-history outcomes

Where childhood adversity was linked to differential paces of ageing, we next examined whether EAA were related to later social and health-related outcomes. Using a multivariable OLS approach, we regressed health related outcomes (BMI z-scores, depression, anxiety) and life-history outcomes (number of children, number of pregnancies) at age 22 on participants’ DunedinPACE scores (measured age 15), comparing models with and without adjustment for earlier adversity exposure at sensitive ages 3 and 9. Coefficient estimates of the DunedinPACE clock are reported for each outcome in Table 3 (see Supplementary Table S4 for full results). Model 1 includes the DunedinPACE clock and a range of covariates (sex, age, education, household income, parental income, race), while Model 2 additionally controls for sensitive ages 3 and 9. Incremental $R^2$ values were additionally reported to quantify the extent to which DunedinPACE contributed to improvements in model fit beyond baseline covariates alone (refer to Supplementary Figure S23 for cross-model comparisons of adjusted $R^2$).

EAA at age 15 was consistently associated with all outcomes in young adulthood (except anxiety), even after accounting for adversity exposure during sensitive periods. An accelerated pace of epigenetic ageing positively predicted increased BMI z-scores in young adulthood, after adjusting for baseline body weight and the two previously identified sensitive periods (*β =0.29*; *se=0.06*; *p<0.001*). Relative to a baseline model including covariates only, including DunedinPACE improved model fit by approximately 30% in Model 1, and 36% after adding both sensitive periods ($\Delta R^2$ = 0.013 and 0.016 respectively) in Model 2. Notably, these incremental gains were largest among all outcomes examined, indicating that EAA contributed most meaningfully in variation in BMI compared to other mental health and life-history domains.

Experiencing accelerated epigenetic ageing was related to increased odds of depression in young adulthood, with each additional year of accelerated ageing being associated with approximately 60% greater odds of experiencing depression (*OR = 1.62*; *se =0.27*; *p <0.05*). Inclusion of both sensitive periods did not attenuate the relationship between DunedinPACE and depression outcomes. However, DunedinPACE contributed only modest improvements in model fit, increasing pseudo-$R^2$ by approximately 8% in Model 1, and 12% after accounting for sensitive periods ($\Delta R^2$ = 0.002 and 0.003 respectively). In contrast, no significant relationship was captured for anxiety outcomes in either models (*OR = 0.9*; *se =0.36*; *p>0.05*), with DunedinPACE producing negligible changes in model fit ($\Delta R^2\le 5\%$).

For life history outcomes, accelerated ageing was positively related to both the number of children ever born, and the number of pregnancies experienced by age 22. Specifically, an accelerated pace of ageing is linked to about 0.22 more children ever born (*β =0.21*; *se =0.08*; *p<0.01*), with increases in model fit of 6% and 11% after including DunedinPACE and sensitive periods ($\Delta R^2$ = 0.004 and 0.007 respectively). A similar pattern emerged for the number of pregnancies experienced by the individual or his partner/spouse, with an accelerated pace of ageing being associated with a greater number of pregnancies even after controlling for both sensitive periods (*β =0.22*; *se =0.11*; *p<0.05*). Including DunedinPACE improved model fit only marginally by about 4% in Model 1, and 8% after adding both sensitive periods ($\Delta R^2$ = 0.002 and 0.006 respectively).

Robustness analyses controlling for cell-type composition showed that EAA only remained predictive of BMI z-scores (*β =0.52*; *se =0.07*; *p<0.001*) and the number of children ever born (*β =0.18*; *se =0.09*; *p<0.05*) after controlling for identified sensitive periods. Full results are shown in Supplementary Table S9.

**Table 3:** Outcome Associations and Model Fit for DunedinPACE.

**Outcome**	**Model Type**	**Model 1**	**Model 2**
BMI z-score	OLS (*β*)	0.29*** (0.06) *Adj. * $R^2 =0.057$ $\Delta R^2 = 29.55\%$	0.29*** (0.06) *Adj. * $R^2 =0.060$ $\Delta R^2 = 36.36\%$
Depression	Logit (OR)	1.62* (0.27) *Pseudo * $R^2 =0.026$ $\Delta R^2 = 8.33\%$	1.62* (0.27) *Pseudo * $R^2 =0.026$ $\Delta R^2 = 12.50\%$
Anxiety	Logit (OR)	0.90 (0.36) *Pseudo * $R^2 =0.020$ $\Delta R^2 <1\%$	0.90 (0.36) *Pseudo * $R^2 =0.021$ $\Delta R^2 = 5.00\%$
NEB	OLS (*β*)	0.22** (0.08) *Adj. * $R^2 =0.067$ $\Delta R^2 = 6.35\%$	0.21** (0.08) *Adj. * $R^2 =0.070$ $\Delta R^2 = 11.11\%$
No. Pregnancies	OLS (*β*)	0.23* (.11) *Adj. * $R^2 =0.082$ $\Delta R^2 = 3.80\%$	0.22* (.11) *Adj. * $R^2 =0.085$ $\Delta R^2 = 7.59\%$

* p<0.05, ** p<0.01, *** p<0.001. OLS coefficients are reported as *β*; Logit coefficients are exponentiated and reported as odds ratios (OR). Incremental $R^2$ values indicate improvement in model fit over the baseline model (sex, age, education, household income, parental income, race). Model 1 adds the DunedinPACE clock to the baseline model, and Model 2 additionally includes sensitive ages 3 and 9.

### Mediating role of EAA in ACE–health and ACE-life history associations

Given the established links between childhood adversity and EAA, and between EAA and subsequent health-related and life-history outcomes, we conducted a mediation analysis to examine whether EAA statistically mediated the relationship between childhood adversity and each outcome of interest (refer to Table 4).

Significant average mediation effects were detected for BMI z-scores (*β =0.003*; *prop* *mediated = 11%*; *p <0.05*), suggesting that associations between ACE exposure and BMI z-scores may partly operate through accelerated ageing. Accounting for the mediator did not result in the loss of significance for the average direct effect (*β =0.023*; $95\%\ CI\ [0.001-0.05]$; *p<0.05*), suggesting that EAA partially mediates the relationship between ACE exposure and BMI. No mediation effects were detected for depression (*β =0.001*; *prop.mediated = 6%*; *p >0.05*) and anxiety outcomes (*β =-0.001*; *prop.mediated = 2%*; *p >0.05*).

In examining life history outcomes, significant mediation effects were observed for both the number of pregnancies (*β =0.002*; *prop.mediated = 4%*; *p <0.05*) and NEB (*β =0.002*; *prop.mediated = 5.6%*; *p <0.05*) outcomes. Mediation effects for life history outcomes were however comparatively weak compared to anthropometric outcomes – EAA only accounted for only approximately 4% and 6% of effects on number of pregnancies and NEB respectively, compared to BMI z-scores (11%).

Sensitivity analyses controlling for epithelial cell composition found little changes to our original results (Supplementary Table S10). EAA still significantly mediated the relationship between childhood adversity and BMI (*β =0.009*; *prop.mediated = 30%*; *p<0.05*) and NEB (*β = 0.0026*; *prop.mediated = 7%*; *p<0.05*), but attenuated in significance for number of pregnancies (*β =0.003*; *prop.mediated = 5.9%*; *p>0.05*). Similar to our main results, mediation by EAA was substantially stronger for BMI z-scores (*prop.mediated = 30%*) compared to NEB (*prop.mediated = 7%*), suggesting that EAA plays a weaker role in influencing outcomes that are more social in nature.

**Table 4:** Mediating Effects of Epigenetic Accelerated Ageing (EAA).

Effect	Path	*β*	95% CI	
			CI Lower	CI Upper
**Outcome: BMI z-score**				
Average Mediation Effect	ACE *→* DunedinPACE *→* BMI z-score	0.003*	0.0005	0.01
Average Direct Effect	ACE *→* BMI z-score	0.023*	0.001	0.05
Total Effect	ACE *→* BMI z-score	0.025*	0.003	0.05
Prop. Mediated		.11		
**Outcome: Depression**				
Average Mediation Effect	ACE *→* DunedinPACE *→* Depression	1.10e-03	1.7e-04	0.00
Average Direct Effect	ACE *→* Depression	1.73e-02	-9.7e-03	0.04
Total Effect	ACE *→* Depression	1.84e-02	8.7e-03	0.04
Prop. Mediated		0.06		
**Outcome: Anxiety**				
Average Mediation Effect	ACE *→* DunedinPACE *→* Anxiety	-0.001	-0.001	0.00
Average Direct Effect	ACE *→* Anxiety	0.01	-0.01	0.03
Total Effect	ACE *→* Anxiety	0.01	-0.01	0.02
Prop. Mediated		0.02		
**Outcome: No. of Pregnancies**				
Average Mediation Effect	ACE *→* DunedinPACE *→* Pregnancies	2.22e-03*	2.2e-04	0.01
Average Direct Effect	ACE *→* Pregnancies	5.32e-02*	1.0e-03	0.10
Total Effect	ACE *→* Pregnancies	5.32e-02*	1.00e-02	0.10
Prop. Mediated		0.04		
**Outcome: NEB**				
Average Mediation Effect	ACE *→* DunedinPACE *→* NEB	0.002*	0.0002	0.01
Average Direct Effect	ACE *→* NEB	0.036*	0.005	0.07
Total Effect	ACE *→* NEB	0.038*	0.007	0.07
Prop. Mediated		0.056		

* p<0.05, ** p<0.01, *** p<0.001

## Discussion

While links between ACEs and EAA in adulthood are well documented^28–30^, this study centres on earlier periods of childhood and young adulthood. We focus on this early life period because it is when biological signals resulting from early adversity are most readily detectable. In contrast, epigenetic ageing observed later in life may be confounded by factors unrelated to childhood adversity, such as negative health behaviours and accumulated biological wear and tear. This study therefore examined how life course exposure to ACEs affects one’s epigenetic age in young adulthood, as well as the role of epigenetic ageing in influencing risky outcomes commonly associated with childhood adversity.

Although the proportion of variance explained by early-life adversity in epigenetic ageing is modest, this magnitude is consistent with prior studies of childhood predictors of DunedinPACE and other epigenetic clocks^31^. These observed small effect sizes suggest that childhood adversity represents one of many factors contributing to the variation in DunedinPACE during development. Furthermore, small effect sizes are expected when examining population-level variation in biological ageing processes, particularly in early life where ageing trajectories are beginning to diverge. Importantly, our aim is not individual-level prediction of ageing but the identification of socially patterned differences in the timing of adversity and DuneinPACE pace of biological ageing.

DunedinPACE differs conceptually from earlier epigenetic clocks commonly used in childhood adversity research. First-generation clocks such as Horvath and Hannum primarily estimate chronological age from methylation profiles^32,33^, whereas second-generation clocks such as PhenoAge^34^ and GrimAge^35^ were developed to capture morbidity and mortality risk using clinical and physiological biomarkers. In contrast, DunedinPACE captures the pace of physiological deterioration across multiple organ systems in midlife^36^. Consequently, findings derived from first- and second-generation clocks may not be directly comparable to those using DunedinPACE, given evidence that earlier clocks are less sensitive to socioeconomic disadvantage, racial marginalisation, and broader health disparities^37,38^. However, studies using earlier-generation clocks demonstrate broader links between social disadvantage in early life and methylation-related biological processes. Our findings should therefore be interpreted specifically in relation to pace-of-ageing processes rather than epigenetic age acceleration more broadly defined.

Our results suggest that *sensitive periods*, rather than *cumulative risk* or *recency*, best accounted for epigenetic ageing patterns in young adulthood. This aligns closely with earlier findings by Dunn et al.,^39^ who discovered sensitive periods of adversity exposure to be most predictive of DNA methylation in adolescence. These results are not surprising, given that biological systems undergo rapid development during their early life stages. Elevated plasticity of the epigenome during this developmental phase may thus explain why adversity experienced at sensitive periods are most strongly related to epigenetic ageing, compared to cumulative risk or recency.

Sensitive periods identified in our analyses varied by the specific types of adversity in question. Exposure to instability and deprivation revealed a sensitive period at age 3; while exposure to poverty showed a sensitive age 9 period. Earlier studies suggest that emotional development during the first three years of life is particularly crucial for shaping children’s later life perceptions and behavioural responses towards stressors^40^. This may explain why we found ACEs related to the emotional growth of a child (e.g., instability and deprivation) to be most influential during the earlier years of their childhood. Contrarily, poverty was shown to matter more for children at later periods of their lives^41^. This coincides closely with our findings, where poverty was found to be most influential during later childhood at age 9. Where no significant associations were detected for maltreatment, this may partly reflect limitations in measurement derived from caregiver reports of abusive behaviours towards the focal child. Social desirability bias may lead to under-reporting by parents^42^, which obscure true associations between maltreatment exposure and DunedinPACE clocks. Our findings therefore represent a conservative estimate of the relationship between maltreatment and one’s pace of epigenetic ageing.

Furthermore, we also found that different kinds of ACEs were associated with varying patterns of epigenetic ageing – instability and deprivation are associated with a *decelerated* pace of ageing, while poverty is associated with an *accelerated* pace of ageing. Collectively, these findings suggest heterogeneity in ageing trajectories according to the type of adversity experienced. This aligns with earlier studies finding stressors related to detrimental physical development (e.g., obesity) to hasten children’s pace of ageing, whereas those related to socioemotional development (e.g., parental depression; harsh parenting) tend to be associated with epigenetic deceleration^43,44^. Thus instability and deprivation ACEs, which relate more heavily to children’s emotional development, may result in a decelerated pace of ageing; in contrast to poverty ACEs heavily linked to the physical growth of children.

The inverse associations observed between instability/deprivation ACEs and the pace of ageing may seem counterintuitive, given substantial evidence linking adversity exposure to accelerated biological ageing^20–22^. Our findings are however neither new nor unexpected. While harm *directly* inflicted on children (e.g., maltreatment and abuse) have indeed consistently produced accelerated pace of ageing, earlier studies have also documented associations between *indirect* threats to children (e.g., household dysfunction) and decelerated ageing as measured by PhenoAge clocks^45^.

Various explanations have been proposed to account for this negative association. Emerging developmental theories suggest that adversity may not uniformly accelerate biological development, but instead recalibrate developmental tempo differentially across developmental contexts^46^. Under this perspective, accelerated and decelerated developmental trajectories are not opposing outcomes, but rather alternative adaptive responses to different forms of adversity. In the context of this study, adversities directly experienced by children may reflect threats that are endangering; while those that are indirectly experienced (e.g., household member mental illness, parental marital disruption) may reflect impaired access to (social/emotional) resources crucial for child development. Previous studies have demonstrated that threatening environments favour accelerated developmental strategies prioritising earlier autonomy and reproduction^47,48^, while limited access to resources and care are linked to developmental slowing or delayed maturation processes until more favourable conditions arise^49^. Decelerated DunedinPACE associations observed for instability and deprivation in our analyses may therefore reflect slower developmental pacing in response to constrained resource environments during childhood.

Alternatively, decelerated epigenetic ageing associated with instability- and deprivation ACEs may reflect downstream effects of parental mental health conditions. Parental psychological disorders during the pre- and antenatal periods are consistently associated with decelerated biological ageing in offspring across multiple epigenetic clocks^43,50,51^. Such impaired parental mental health is also linked to marital disruption and poorer parent–child interactions^52,53^, which we proxy using instability and deprivation ACEs. These ACEs may therefore reflect pathways through which pre-birth parental mental health influences decelerated epigenetic ageing, though we are unable to test this directly due to the lack of pre-birth data in the FFCWS. Hence, such patterns should not be interpreted as inherently health-protective or indicative of slower biological ageing in a normative sense.

Next, we explored associations between the DunedinPACE clock in relation to anthropometric, mental health, and life-history outcomes in young adulthood. We find that children with EAA were more likely to experience greater *BMI, depression risk, NEB*, and *number of pregnancies*, net of effects exerted by adversity exposure at sensitive periods. Where prior research has reported contemporaneous associations between age-15 DunedinPACE, psychological functioning and mental health within the Future Families cohort^54^, our study extends these findings by demonstrating the persistent association between age-15 DunedinPACE and downstream outcomes measured at age 22. Associations with mental health outcomes however appeared more selective in young adulthood, with significant associations observed for depression risk but not anxiety. This pattern may suggest that some contemporaneous associations between DunedinPACE and mental health weaken over longer developmental periods or vary across mental health domains. These findings suggest that an accelerated pace of ageing in adolescence may have implications extending beyond contemporaneous psychological functioning, shaping subsequent health-related and developmental trajectories into young adulthood.

Where associations between EAA and health outcomes have been heavily documented in earlier literature^16,55^, much less is known about life-history strategies relating to childbearing outcomes. Earlier studies have focused on sexual behaviour in adults, such as one’s age at sexual debut and number of sexual partners^56,57^, but none have examined actual childbearing outcomes in young adulthood. Unlike sexual behaviours which may not lead to actual childbearing, actual childbearing outcomes reflect an early commitment to reproductive efforts rather than just sexual experimentation.

Our findings provide evidence that age-15 EAA is related to an increased number of children ever born, and pregnancies experienced by individuals at a relatively young age (22 years old). Although reproductive outcomes are strongly shaped by broader social and structural contexts^58^, these findings remain broadly consistent with life-history perspectives proposing that exposure to harsh or unstable environments may be associated with a hastened life-history strategy, evidenced by earlier reproductive investment and developmental acceleration^47,48^. Our findings therefore suggest that epigenetic ageing may represent one biosocial correlate of reproductive trajectories embedded within broader developmental and structural contexts.

From a life-history perspective, accelerated reproductive timing reflects a broader developmental trade-off where energetic resources are channelled towards earlier reproduction, rather than long-term somatic maintenance and health preservation^3,4^. An accelerated pace of ageing may therefore capture one dimension of developmental resource allocation under adversity, whereby individuals exposed to harsh or unstable early-life environments shift toward faster developmental and reproductive trajectories.

Finally, we examined whether EAA statistically mediated the relationship between childhood adversity and our outcomes of interest. We find significant mediation for BMI z-scores, but not mental health outcomes. Significant, but weak mediation coefficients were also detected for life-history outcomes such as early adulthood fertility behaviours. This suggests that EAA may be more closely associated with pathways linked to anthropometric and physical health-related outcomes, rather than psychological or ‘social’ outcomes. The relatively weak or absent mediation effects for mental health and life-history outcomes further suggest that other psychological, social, and environmental processes may also contribute importantly to these associations beyond epigenetic ageing, underscoring the complexity of how early-life stressors are associated with different aspects of health and behaviour across the life course.

Our findings underscore the heterogeneous pathways through which childhood adversity becomes biologically embedded over the life course. Building on studies evidencing the deleterious consequences of childhood adversity, we show that the timing of exposure—particularly during sensitive developmental periods—may play a disproportionate role in shaping later health outcomes and life-history trajectories. Consistent with studies linking EAA to adverse health and developmental outcomes^15,16^, results show that EAA partially mediates the association between childhood adversity and BMI z-scores and life-history traits. These results highlight epigenetic ageing as a potential biosocial mechanism linking early-life adversity to long-term physiological and developmental consequences. Future research should further address the pathways through which childhood adversity influences epigenetic ageing, and assess how these processes contribute to health deterioration and accelerated life-history strategies across the life course.

Our study offers three notable contributions. First, we build on existing empirical applications of the life course perspective by examining how the *timing* of exposure to childhood adversity shapes future biological and social consequences. By explicitly testing competing life course models, we contribute to life-history theory by demonstrating that adversity exposures at different developmental stages are related to distinct and lasting influences on later life health and reproductive trajectories.

Second, our study is the first to examine epigenetic ageing as a mediator linking childhood adversity to health and life-history outcomes. By demonstrating that epigenetic age acceleration statistically mediated associations between adversity and subsequent health-related and life-history traits, we provide evidence that epigenetic ageing serves as a potential pathway through which socially experienced stress becomes biologically embedded. These findings highlight how early-life experiences become biologically embedded, influencing both epigenetic ageing and downstream social and reproductive outcomes. This underscores the importance of considering both biological and social processes in understanding the long-term consequences of childhood adversity.

Third, our findings contribute to broader discussions regarding developmental vulnerability and the timing of adversity exposure during childhood. Existing child protection services, for example, are highly dependent on self-reports by children or suspecting third-parties^59^. Yet, children in our identified sensitive periods (ages 3 and 9) often possess more restrictive institutional resources to recognise and report adverse experiences, potentially reducing the visibility of adversity exposures during early developmental periods. Although the present findings should not be interpreted as definitive developmental windows for intervention, they underscore the broader importance of early childhood environments in shaping later developmental and health-related outcomes. Future research may further clarify how developmental timing interacts with social and institutional contexts to influence vulnerability across childhood.

Together, our findings demonstrate that biological ageing processes are already socially patterned in adolescence, and that the timing of adverse exposures may be more consequential for epigenetic ageing than cumulative burden alone. Applying a structured life course modelling approach to epigenetic ageing, this study shows that divergence in ageing trajectories may emerge well before adulthood, highlighting childhood as a potentially sensitive developmental period for the social shaping of ageing. These results underscore the importance of integrating life course perspectives into ageing research, and suggest that interventions aimed at reducing early-life adversity may have implications not only for immediate wellbeing but also for long-term biological ageing processes.

## Data and methods

### Data

Data was derived from the Future of Families and Child Wellbeing Study (FFCWS). The FFCWS utilises a stratified multistage sampling approach, comprising a sample of 4,898 children born in the U.S. between 1998-2000 and their families. We focus on the U.S. because of its remarkably high prevalence of childhood adversity: about 25% of American children experienced childhood adversity before age 4, and approximately 62.8% of adults report being ever exposed to ACEs^60^. Births to unmarried mothers were intentionally oversampled at a ratio of 3 to 1, ensuring comprehensive representation across various demographic groups, including Black, Hispanic, and low-income households. The FFCWS follows participants across 7 waves, from the focal child’s birth until age 22. Further information is provided within the FFCWS public documentation^61^. Ethical approval for this research was granted by the Research Ethics Committee of the Department of Sociology (DREC) at the University of Oxford. All methods were performed in accordance with relevant institutional guidelines and regulations and in accordance with the Declaration of Helsinki. Informed consent was obtained from all participants by the original data collectors^61^.

We utilised data from relevant surveys to answer our research questions: (1) primary caregiver (PCG) survey; (2) core mother’s survey; (3) core father survey; (4) child survey; and (5) biomarker data. PCG surveys collected from wave 3 onwards comprise data about the child’s upbringing (e.g., disciplinary measures; parent-child relationship), whereas core mother and father’s surveys contain data pertaining to the personal circumstances of both parents (e.g., legal troubles; substance abuse). The child survey covers the personal circumstance of the focal child, such as mental health and educational attainment. Saliva DNA and biomarker data are collected in waves 5 and 6, comprising information such as epigenetic clocks and DNA methylation.

Characteristics of our analytic sample are presented in Table 5. We have approximately equal proportions of males and females, and the sample is predominantly composed of non-white children (88.22%) with non-white mothers (77.46%). Most mothers did not attend or graduate from college (61.85%) (Table 6).

**Table 5:** Descriptive Statistics of Analytic Sample.

		Total		DunedinPACE		Cumulative Risk		Recency	
		N	%	Mean	SD	Mean	SD	Mean	SD
Sex	Female	982	49.75	1.29	0.18	2.68	2.08	14.92	11.91
	Male	992	50.25	1.24	0.18	2.69	2.05	15.03	11.95
Race	Non-white	1623	82.22	1.28	0.18	2.91	2.04	16.21	11.87
	White	351	17.78	1.20	0.17	1.64	1.80	9.28	10.44
Maternal Race	Non-white	1529	77.46	1.28	0.18	2.91	2.04	16.17	11.84
	White	445	22.54	1.20	0.17	1.91	1.95	10.87	11.33
Maternal Education	Degree	753	38.15	1.25	0.18	1.81	1.85	10.08	10.65
	No degree	1221	61.85	1.28	0.18	3.23	2.00	18.00	11.67

**Table 6:** Distribution of ACE types by time point.

		Age 3		Age 5		Age 9	
		Mean	SD	Mean	SD	Mean	SD
Sex	Female	0.96	0.93	0.86	0.86	0.86	0.89
	Male	0.94	0.92	0.89	0.87	0.86	0.90
Race	Non-white	1.03	0.92	0.95	0.87	0.93	0.90
	White	0.58	0.83	0.51	0.73	0.56	0.80
Maternal Race	Non-white	1.03	0.92	0.96	0.88	0.92	0.90
	White	0.65	0.87	0.60	0.77	0.66	0.85
Maternal Education	Degree	0.64	0.86	0.59	0.78	0.58	0.81
	No degree	1.14	0.92	1.05	0.87	1.04	0.90

### Measures

**Fig. 2:**
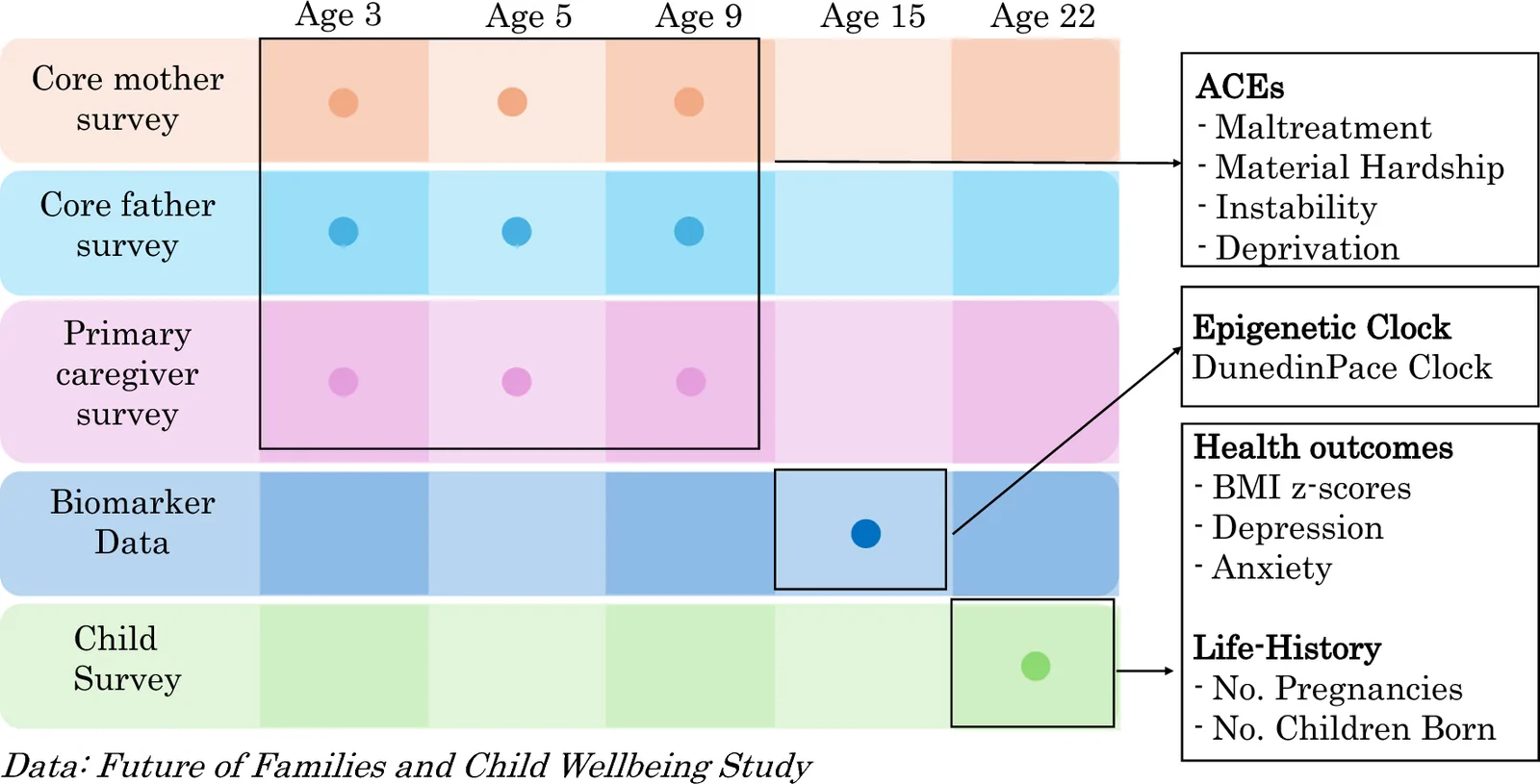
Key study measures across waves.

Figure 2 below presents a visual schematic of the key independent and dependent measures across study waves in the FFCWS. The figure summarises the timing of childhood adversity exposures, the assessment of epigenetic clock, and the measurement of young adult health and life-history outcomes.

#### Epigenetic clock measure

Saliva samples were collected by Westat Inc., the subcontractor for the FFCWS, during waves 5 and 6 (ages 9 and 15) of the home visit survey. Saliva samples were collected using the Oragene DNA Self-Collection kits by trained interviewers. Participants who were not part of the home visit survey received and returned the saliva collection kits via mail. In total, 2,884 unique child samples and 2,670 unique mother samples were collected, and underwent analysis using the Illumina Infinium Human Methylation450K (450K) or Illumina Infinium MethylationEPIC (EPIC) array in line with the manufacturer’s guidelines. Quality control of the methylation data was performed using the *EWAStools* R package^62^. Samples were excluded if the detection value was larger than 0.01 and 0.05 for the 450K and EPIC arrays respectively. Probes were also removed if the number of methylated or unmethylated bead count was fewer than four. Using the *ENmix* R package procedure, probes were also excluded if they were flagged for outlier methylation or bisulfite conversion values, or sex mismatches between recorded data and methylation predictions.

Cell-type proportions were estimated using four reference panels. The EpiDISH method estimates the proportions of epithelial, fibroblast, and immune cells based on predefined cell-type signatures^63^. The child saliva panel is based on the Middleton reference panel for saliva, available in the *EWAStools* R package^64^, and includes estimates of epithelial and immune cell proportions derived from sorted saliva samples from children aged 7–16 years. The blood immune cell panel uses the Salas reference panel, also implemented in *EWAStools*, to estimate the relative abundance of specific immune cell types in blood, including granulocytes, natural killer cells, B cells, CD4 and CD8 T cells, and monocytes^65^. Finally, select immune cell types were estimated using the Horvath method, which provides ordinal abundance measures for plasma blasts, CD8+ CD28- CD45RA- T cells, and naïve CD8 T cells^66^.

We focus on the wave 6 DunedinPACE epigenetic clock as our main dependent variable in this study. The DunedinPACE clock is based on genome-wide methylation profiles generated by the the Illumina Infinium Human Methylation450K (450K) or Illumina Infinium MethylationEPIC (EPIC) array. Detailed information about the generation of epigenetic clocks is provided in the FFCWS public documentation^67^. To reduce the number of missing cases in our analytical sample, epigenetic clock data generated by both Illumina 450K and EPIC arrays were combined, in line with recommendations in the literature^68,69^. A positive value of the DunedinPACE clock indicates an *accelerated* pace of epigenetic ageing (i.e., that one is biologically older than his/her chronological age), while a negative value indicates the contrary^36^. A DunedinPACE value of 0 reflects that an individual’s epigenetic age directly corresponds with his chronological age – i.e., that he/she is not experiencing accelerated or decelerated ageing. We dropped observations with missing epigenetic clock data from the analysis, yielding a final analytic sample of 1,974 individuals.

#### Adverse childhood events measures

Data relevant to adverse childhood experiences were obtained from parent-specific and PCG surveys. These surveys collect information pertaining to parents’ economic and employment status, parenting behaviour, relationship status, and other information. Pertinent to this study are data related to the adverse early household and upbringing conditions experienced by children. We examine four domains of childhood adversity: *Childhood maltreatment; Material Hardship; Early Life Instability; and Familial Deprivation*. Data relevant to these ACEs were extracted from waves 3, 4, and 5 (corresponding to ages 3, 5, and 9) of the parent-specific and PCG survey. Wave 2 (age 1) data was excluded from analysis due to differences in survey instruments accessing ACEs.

*Childhood maltreatment* measures the extent to which a child was subjected to abusive behaviour (e.g., shaking or slapping child) as reported by their PCGs. Parents and PCGs were asked, for instance, if they had hit the child with a belt or other objects; or if they swore at the focal child in the past year. Children were treated as being exposed to maltreatment if PCGs reported any abusive behaviours at all.

*Poverty* measures if a child has suffered socioeconomic disadvantage in their childhood as indicated by their poverty ratio. The poverty ratio is calculated by taking household incomes as a fraction of family-composition specific poverty threshold established by the US Census Bureau. Children were treated as having experienced poverty if mothers reported being in poverty (i.e., poverty ratio < 1). *Early life instability* examines if a child faced instability and changes linked to their familial environments. We adopt the measure utilised by Doom et al.,^70^ which considers changes in maternal romantic status across time. Children were treated as experiencing early life instability for that wave if the romantic relationship status of their mother was different from that one wave earlier.

The final ACE domain –family deprivation– represents if a child was exposed to parenting styles associated with a lack of affection or emotional unavailability. In line with Doom et al.^70^, this is measured through variables related to the frequency of mother-child interactions (e.g., reading or singing to child; showing physical affection to child). Children are treated as being exposed to this ACE if mothers reported not engaging with their children at all.

We outline the distribution of adversity events across the measured time points. Table 7 presents the proportion of the sample exposed to each type of adversity at different ages, whereas Table 8 displays the distribution of the number of ACEs experienced by the sample across time points. Poverty appeared to be the most prominent form of adversity experienced by children across all three time points (approximately 40%), whereas maltreatment were the least reported (approximately 15%). Table 9 summarises the intraclass correlation coefficients (ICCs) for each adversity type, indicating the stability of exposure across childhood. Higher ICC values reflect greater consistency within individuals over time, while lower values suggest more variability in adversity exposure across ages. Thus, poverty (ICC = 0.476) and maltreatment (ICC = 0.259) appears to be the most stable within individuals across time, as compared to instability (ICC = 0.119) and deprivation (ICC = 0.111).

**Table 7:** Distribution of ACE types by time point.

ACE Type	Age 3	Age 5	Age 9
Poverty	786 (39.8%)	773 (39.2%)	718 (36.4%)
Instability	370 (18.7%)	343 (17.4%)	317 (16.1%)
Deprivation	171 (8.7%)	176 (8.9%)	435 (22%)
Maltreatment	276 (14%)	259 (13.1%)	304 (15.4%)
Observations	1,974	1,974	1,974

**Table 8:** Distributions of ACE counts by time point.

ACE Count	Time 1	Time 2	Time 3
0	842 (42.7%)	845 (42.8%)	769 (39%)
1	749 (37.9%)	771 (39.1%)	756 (38.3%)
2	305 (15.5%)	297 (15%)	339 (17.2%)
3	68 (3.4%)	58 (2.9%)	100 (5.1%)
4	10 (0.5%)	3 (0.2%)	10 (0.5%)
Observations	1,974	1,974	1,974

**Table 9:** ICC estimates of ACE stability by time point.

ACE Type	ICC	Lower CI	Upper CI	P-value
Poverty	0.476	0.450	0.502	< 0.001
Instability	0.119	0.092	0.147	< 0.001
Deprivation	0.111	0.082	0.139	< 0.001
Maltreatment	0.259	0.230	0.288	< 0.001

#### Health and life history measures

Health and life-history outcomes in young adulthood are the secondary outcomes of interest in this study. We retrieve relevant information from the wave 7 (age 22) Young Adult Survey in the FFCWS, which were administered to the focal child in one of three modes: (1) self-administered online platform; (2) phone interview; or (3) in-person survey. Anthropometric outcomes are measured using BMI z-scores, which are consistently linked with mortality and disease risk in later life^71–73^. Mental health outcomes are proxied by depression and anxiety status, which are derived from the *Composite International diagnostic Interview - Short Form (CIDI-SF)* completed by focal children. The CIDI is consistent with the Diagnostic and Statistical Manual of Mental Disorders - Fourth Edition (DSM-IV). The short form of the CIDI instrument takes a portion of the full set of questions, and calculates for each child the probability of being considered a *case* (i.e., diagnosed with mental health condition) if given the full interview^74^. The focal child is treated as depressed/anxious if the score is above 0.5. Life history outcomes are measured by: 1) the number of pregnancies a focal child or his partner has had; and 2) the number of children ever born (NEB) to the focal child by age 22 (wave 7).

#### Covariates

We include focal child’s age, race, and gender as potential confounders because they are highly correlated with both childhood adversity and epigenetic ageing trajectories^75,76^. In line with existing literature, we also control for other potential confounders, including mother’s race, mother’s age at childbirth, and mother’s education levels^31^. Maternal traits were dominantly treated as potential confounders because they serve as the primary caregivers of the focal child, and their characteristics have been shown to significantly influence children’s ACE exposure and biological outcomes^31^. In examining children’s health and life-history outcomes, socioeconomic measures such as parental household income, child’s educational level, and child’s household income, were also included as covariates. All covariates were taken from the child, parent-specific, and PCG surveys.

### Statistical methods

#### Structured life course modelling approach

We examine three life course hypotheses in this study: *cumulative risk; recency; and sensitive periods*. The foremost hypothesis is represented by a cumulative risk index calculated by summing together all ACE events across all waves. The second model is measured through a recency index, by weighting year-specific ACE events by the age at ACE exposure. Thus, if children were exposed to 1 ACE during year 1 (wave 2 of the FFCWS), and 2 ACEs during year 9 (wave 5), then said child would have a recency score of $(1\times 1) + (2\times 9) =19$. The sensitive period model is represented by year-specific counts of ACEs that children were exposed to. Scores for these three life course models were also calculated for each of the four ACE domains. The three hypotheses are summarised in Table 10.

**Table 10:** Operationalisation of Tested Life Course Models.

**Life Course Model**	**Definition**	**Calculation Method**
Cumulative Risk	Total burden of multiple risk factors or adverse experiences over time	Sum of ACE exposures across all years and domains: Cumulative Score = $\sum \limits _{w=3}^{5} ACE_w$
Recency	Disproportionate effects exerted by the recency of adversity exposure	Sum of ACE exposures weighted by focal child age: $Recency\ Score = \sum \limits _{w=3}^{5} ACE_w \times Age_w$
Sensitive Period	Disproportionate effects exerted by adversity exposure at particular ages during development	Number of ACE exposures within each wave: $Sensitive\ Score = ACE_w$

We differentiate between the various life course models using a structured life course modelling approach (SCLMA). SCLMA adopts a two-step procedure to select the most optimal life course model that accounts for variation in EAA across individuals^39,77,78^. In the first step, we adjusted our life course models for confounding by residualising them with respect to covariates. These residualised life course models were then fitted into a least angle regression (LARS). Analogous to traditional stepwise regression, the LARS algorithm incrementally builds a parsimonious model by adding variables based on their correlations with the residuals. A predicted value ( $\hat{y}$ ) is calculated for each iteration, and subtracted from the observed response variable (*y*). The LARS procedure occurs iteratively, and terminates after the number of steps needed to minimise the $C_p$ statistic, as given by the following:

$$\displaystyle {C_p = (1 / \hat{\sigma }^2) \sum _{i=1}^{n} (y_i - \hat{y}_i)^2 - n + 2k}$$

where *n* equals the number of observations, *k* represents the number of steps taken, and $\hat{\sigma }^2$ represents the estimated residual variance. The resulting variables selected then constitute life course models that most strongly describes the relationship between the exposure and outcome.

Where the first stage of the SCLMA procedure fits the LARS model with the three life course hypotheses, the second stage is concerned with selecting the most parsimonious model produced by SCLMA. Here, we adopt two complementary approaches. First, an elbow plot is produced to identify the optimal number of predictors (i.e., life course models), based on improvements in $R^2$ values. The chosen model is typically one in which the ‘next best’ model yields declining marginal returns in its $R^2$ value. This is represented by a sharp decline in the elbow plot, indicating little improvement in the $R^2$ value for the subsequent life course model. Complementarily, we also applied post-selection inference by considering the p-values of life course models suggested by the elbow plots. We sequentially removed non-significant predictors according to p-value sizes, and only retained life course models if they remained significant after adjusting for covariates in an OLS regression. The final set of predictor(s) models then represents the life course models that most saliently predicted EAA in later life. If the optimally selected model is one which involves 2 (or more) variables, then a compound hypothesis combining multiple life course frameworks is the best proposed model explaining the exposure-outcome relationship.

Although LARS is shown to produce biased estimates when exposure variables are highly correlated (*r > 0.9*)^78^, we demonstrate that our study variables do not share strong correlations (Supplementary Table S1), and are therefore unlikely to introduce bias into our estimates (*r=0.12-0.52*).

#### OLS and mediation analysis

To answer our second research question, we conducted two multivariable regression analyses to examine if EAA predicts health-related and life-history related outcomes in young adulthood. The first model regresses the DunedinPACE clock on each outcome of interest with controls for potential confounders; while the second model further controls for the life course model identified earlier. This investigates if EAA still predicts health and life-history related outcomes after accounting for the child’s exposure to adversity.

We also employ mediation analyses to answer our third research question, to examine if EAA mediates the relationship between ACE exposure and health/life-history related outcomes. We estimate the direct and indirect effects of ACE exposure on health/life-history related outcomes, and the proportion of effects mediated through EAA. 95% CIs were generated through non-parametric bootstrapping with 1,000 resamples. The full analytical pipeline is presented in Figure S1 of the Supplementary Information.

#### Sensitivity analyses

To assess the robustness of our life course model selection, we conducted several sensitivity analyses. First, to differentiate the *recency* model from the latest age-9 *sensitive period* hypothesis, we adopted two alternative specifications of the *recency* model. The first specification defines recency as the *time difference* between age 15 (when the epigenetic clock was derived) and the age at last ACE exposure; whereas the second specification further weighted this measure by the number of ACEs at last exposure. Secondly, we re-operationalised the *cumulative risk* model by collapsing respondents with three or more adversities into a single category, due to the low frequency of individuals experiencing such high levels of childhood adversity. The SCLMA procedure was then refitted with these revised measures to evaluate whether the selected life course models remained consistent with those identified in the initial analysis.

To address potential confounding from cell-type heterogeneity, epithelial cell composition was included as a covariate to evaluate whether the associations between the selected life course models and EAA were attenuated. Cell composition was likewise controlled for in models regressing health and life-history outcomes on EAA, as well as in the mediation analyses. As cell-type composition may also operate as an mediating variable (rather than a confounder), follow-up analyses were conducted to test whether cell composition mediated the association between adversity exposure and subsequent EAA, to assess the appropriateness of its inclusion as a covariate.

However, our primary analyses are designed to estimate the total association between early-life adversity and epigenetic ageing. Adjusting for epithelial cell composition would address a different causal estimand, as cell-type proportions may lie on the pathway through which adversity becomes biologically embedded. Conditioning on such variables could therefore remove meaningful components of the total effect. For this reason, we present models without adjustment for epithelial cell composition as our main specification, while additional analyses incorporating cell-type adjustment are provided to illustrate the sensitivity of results to alternative modelling choices. This distinction aligns with recent cautions regarding overadjustment in epigenetic epidemiology^79^.

## Study limitations

Within the context of our study, the sensitive period hypothesis suggests that effects of childhood adversity on epigenetic ageing are driven solely by differences in exposure *timing*. This assumes that adversities, as well as the contexts under which they occur, remain constant throughout the observed time span. However social and economic circumstances, as well as other childhood conditions may improve or deteriorate across time. Hence, there may be cross-wave variability in how childhood adversity is measured and experienced. To address this, we presented data on the distribution of adversity exposure across waves, and intraclass correlation coefficient (ICC) estimates for ACE measures. While overall ACE distributions remained relatively similar across developmental waves (Table 7), intraclass correlation coefficient estimates indicated varying levels of temporal stability across adversity domains, with poverty demonstrating substantially greater stability than instability or deprivation (Table 8). This suggests that some observed timing effects may partly reflect differences in the measurement consistency of adversity exposures across childhood. Additionally, selective attrition may be present if individuals exposed to more severe adversity were differentially retained over time. The capacity for children to respond to stressors-may also grow as they age. This may partly account for the salience of early- rather than later sensitive periods for some domains of adversity in our study.

Additionally, the present study’s analytical design is observational and cannot derive causal conclusions, despite the temporal ordering of adversity exposure, DunedinPACE, and later outcomes. Although we adjusted for a broad range of sociodemographic covariates, the present analyses cannot account for all factors potentially shaping health-related or reproductive outcomes in young adulthood. For instance, reproductive outcomes in young adulthood are influenced by various intersecting socio-structural conditions, such as neighbourhood conditions and socioeconomic opportunities. Residual confounding from these unmeasured factors may therefore partly contribute to the observed associations between childhood adversity, epigenetic ageing, and reproductive outcomes. Findings should therefore be interpreted as identifying longitudinal associations consistent with biological embedding processes rather than causal pathways. Future studies should incorporate richer measures of educational opportunities, relationship contexts, and structural inequalities accompanied by quasi-experimental designs to disentangle biological embedding processes from broader socio-structural pathways shaping reproductive and health-related outcomes.

Indicators of adverse childhood experiences in the present study relied on parental and caregiver self-report measures, including reports of verbal and physical maltreatment. Such measures may be vulnerable to under-reporting and social desirability bias^42^, particularly for highly sensitive parenting behaviours. Measurement limitations of this kind may attenuate observed associations between childhood adversity exposures and DunedinPACE, potentially contributing to weaker or null findings for certain ACE domains.

## Supplementary Information

Supplementary Information.

## Data Availability

The data for this study can be obtained from the Fragile Families and Child Wellbeing Study website (https://fragilefamilies.princeton.edu/). Detailed information on how to access and utilise the dataset can be found on their official website.

## Code availability

All code used to produce the results presented in this paper, as well as the statistical results themselves are available in the Supplementary Information and are available at: https://github.com/wesleywj/AdverseChildhood_EpigeneticAgeing.
