# Supplementary material for: Timing of adverse childhood experiences shapes epigenetic ageing and life-history outcomes
Source: Sci Rep. 2026 Jun 19;16:28031. doi: 10.1038/s41598-026-58459-1 (PMC13549978; doi:10.1038/s41598-026-58459-1)
Supplement: Supplementary file 1 [file 41598_2026_58459_MOESM1_ESM.pdf]

Supplementary Information

Analytical Framework

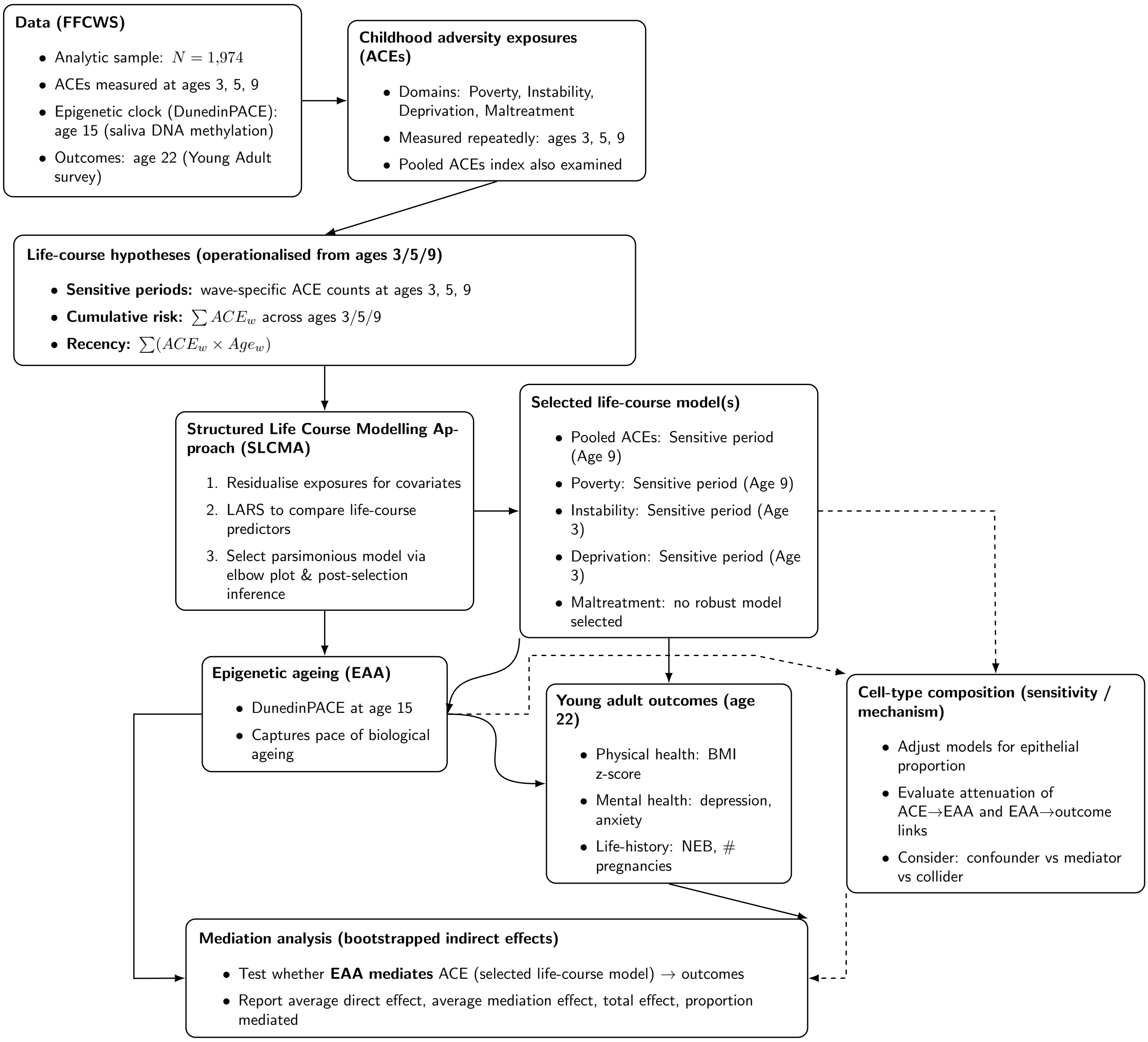

Figure S1: Conceptual framework of study

Structured Life Course Modelling Approach

The structured life course modelling approach adopts a two-step procedure to select the most optimal life course model that accounts for variation in EAA across individuals. The first step comprises a least angle regression (LARS). Analogous to traditional stepwise regression, the LARS algorithm incrementally builds a parsimonious model by adding variables based on their correlations with the residuals, without imposing shrinkage in the same way as penalised methods like Lasso or Ridge regressions.

Procedurally, the LARS procedure starts off with a model where all coefficients of predictor variables are assumed to be zero, with only an existing intercept (β_0). A predicted value ($\hat{y}$) is obtained from this intercept, and a residual r is calculated by subtracting the $\hat{y}$ from the observed response variable (y). Next, a predictor variable most correlated with the residual, say X_{j1}, is selected and inserted into the model. As before, a predicted value $\hat{y}$ is computed from the newly selected predictor ($\hat{y} = \gamma X_1$), and a residual is obtained by deducting the updated $\hat{y}$ values from the observed y values.

$$r = Y - \gamma X_1$$

The coefficient γ is then increased from 0 to a certain value until a second predictor, say X_{j2}, has just as much correlation with residual r relative to X_{j1}. This second predictor then enters the model, and an updated $\hat{y}$ is obtained and subtracted from y. As usual, the coefficient of this second predictor, γX_2, is then updated until a third predictor variable of equal correlation to the residual is identified, and so on. The LARS procedure is then terminated after the number of steps needed to minimise the C_p statistic, as given by the following:

$$C_p = (1/\hat{\sigma}^2) \sum_{i=1}^{n} (y_i - \hat{y}_i)^2 - n + 2k$$

where n equals the number of observations, k represents the number of steps taken, and $\hat{\sigma}^2$ represents the estimated residual variance. The resulting variables selected then constitute the most parsimonious model that describes the relationship between the predictor(s) and outcome variable.

Thus, the above procedure can summarised pictorially in the illustration below. Assume a scenario with two predictor variables, $X1$ and $X2$. Point O represents the prediction $\hat{y}$ initially, when all coefficients are set to 0 with only an intercept present in the model. From here on, assume that the model identifies $X1$ as the variable most correlated with the residual. $X1$ then enters the model, and the coefficient γX_1 is increased from 0 to a certain value in the direction of Point A. Beyond a certain point, say point B, another variable ($X2$) will equal the correlation between $X1$ and the response variable, and thus inserted into the model. From here on, the LARS regression will then 'shift' from path A to path C, where line C is the predicted value $\hat{Y} = \gamma_1 X_1 + \gamma_2 X_2$. Thus, point B can be said to be the point at which line BC will bisect the angle ABD, resulting in angle DBC being equiangular to angle ABC. Coefficients present in the model are then increased from 0 to certain value, until a third variable is found to be equally correlated with the residual and inserted into the model, and so on.

Where the first stage of the SCLMA procedure fits the LARS model with the three life course hypotheses, the second stage is concerned with selecting the 'best' model

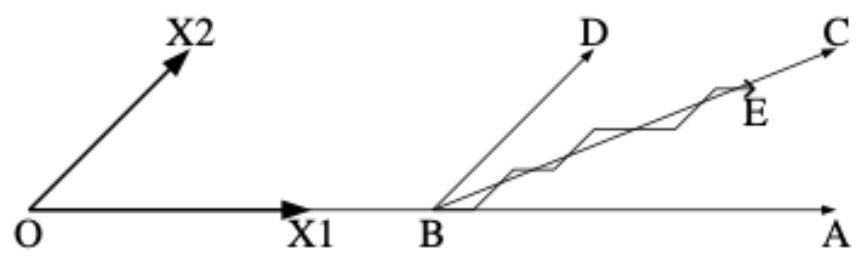

produced by SCLMA. Here, we adopt two complementary approaches. First, an elbow plot is produced to identify the four best models, as well as the improvements in R^2 values associated with each model. The chosen model is typically one in which the 'next best' model yields declining marginal returns in its R^2 value. This is often represented by the model in which a sharp decline in the elbow plot is observed, indicating little improvement in the R^2 value for the subsequent life course model. If the optimally selected model is one which involves 2 (or more) variables, then a *compound hypothesis* combining multiple life course frameworks is the best proposed model explaining the exposure-outcome relationship.

A complementary approach applies post-selection inference methods to choose the best model based on statistical criteria such as effect estimates, standard errors and p-values. The adoption of post-selection inference methods is crucial because model selection based simply on the elbow plot is inadequate to establish if the resulting parsimonious model is indeed significantly predictive of EAA. Post-selection inference methods can be used to derive unbiased estimates because it accounts for multiple hypothesis testing that occurs during the LARS procedure. Various post-inference methods, such as the covariance test and selective inference, are frequently deployed in studies. We adopt the selective inference method in this study as the primary selection criteria, given its ability to yield moderate statistical power and minimise the familywise type I error rate.

Table S1: Correlation across ACE domains and years

	**Poverty**			**Instability**		
Age	Year 3	Year 5	Year 9	Year 3	Year 5	Year 9
Year 3	1	-	-	1	-	-
Year 5	0.52	1	-	0.17	1	-
Year 9	0.44	0.47	1	0.07	0.12	1
	**Maltreatment**			**Deprivation**		
Age	Year 3	Year 5	Year 9	Year 3	Year 5	Year 9
Year 3	1	-	-	1	-	-
Year 5	0.30	1	-	0.16	1	-
Year 9	0.22	0.26	1	0.08	0.13	1

Table S2: Life course model selection (Full Model)

	DunedinPACE Ageing				
	Pooled ACEs	Poverty	Instability	Deprivation	Maltreatment
Constant	1.27**	1.27**	1.29**	1.28**	1.28***
	(0.02)	(0.02)	(0.02)	(0.02)	(0.02)
**Life Course Models**					
Sensitive Period (Age 3)			**-0.027****	**-0.033****	0.011
			(0.010)	(0.014)	(0.012)
Sensitive Period (Age 9)	**0.012***	**0.021***	0.011		0.009
	(0.005)	(0.009)	(0.011)		(0.011)
**Covariates**					
Male	-0.046**	-0.045**	-0.047**	-0.046**	-0.047***
	(0.008)	(0.008)	(0.008)	(0.008)	(0.008)
White	-0.035	-0.035	-0.040*	-0.038*	-0.038*
	(0.018)	(0.018)	(0.018)	(0.018)	(0.018)
White Mother	-0.048**	-0.047**	-0.046**	-0.048**	-0.047***
	(0.017)	(0.017)	(0.017)	(0.017)	(0.017)
Non-Degreed Mother	0.017	0.014	0.020*	0.021*	0.020*
	(0.009)	(0.009)	(0.009)	(0.009)	(0.009)
Maternal Age at Birth	0.0007	0.0006	0.0005	0.0006	0.0007
	(0.0007)	(0.0007)	(0.0007)	(0.0007)	(0.0007)
Observations	1,974	1,974	1,974	1,974	1,974
R^2	0.060	0.060	0.060	0.059	0.058
Adjusted R^2	0.057	0.057	0.057	0.057	0.054

* p<0.05, ** p<0.01, *** p<0.001

All life course models selected by the LARS procedure were initially fitted into a multivariable OLS regression shown above. Life course models that were found to be non-significant were sequentially removed and re-fitted until only significant life course models remained. For the pooled ACEs model therefore, we first regressed the DunedinPACE clock on the life course models identified by LARS –*sensitive age 3* and *sensitive age 9*– while controlling for potential confounders. We then removed the sensitive age 3 life course model due to its non-significance, and refitted the model. We repeated this procedure for all individual ACE domains.

Primarily, our LARS procedure identified various life course candidate models across all ACE domains. Where only an age 9 sensitive period was identified for poverty-related ACEs, both age 3 and 9 sensitive periods were also selected for instability-related ACEs. The age 9 sensitive period was however found to be non-significant, and therefore dropped from the model. Similarly, ages 3, 5, and 9 were identified as sensitive periods of exposure for deprivation-related ACEs. We subsequently dropped ages 5 and 9 in view of their lack of predictiveness. Maltreatment-related ACEs were initially found to have

a sensitive period of ages 3 and 9, although both were eventually dropped due to their non-significance.

Table S3: Life course model selection (Selected Model)

	DunedinPACE Ageing				
	Pooled ACEs	Poverty	Instability	Deprivation	Maltreatment
Constant	1.27**	1.27**	1.29**	1.28**	1.28**
	(0.021)	(0.021)	(0.021)	(0.021)	(0.021)
**Life Course Models**					
Sensitive Period (Age 3)			**-0.027****	**-0.032***	0.011
			(0.010)	(0.014)	(0.012)
Sensitive Period (Age 9)	**0.011***	**0.021***			0.009
	(0.005)	(0.009)			(0.011)
**Covariates**					
Male	-0.046**	-0.045**	-0.047**	-0.046**	-0.047**
	(0.008)	(0.008)	(0.008)	(0.008)	(0.008)
White	-0.035	-0.035	-0.041*	-0.037*	-0.038*
	(0.018)	(0.018)	(0.018)	(0.018)	(0.018)
White Mother	-0.048**	-0.047**	-0.045**	-0.049**	-0.047**
	(0.017)	(0.017)	(0.017)	(0.017)	(0.017)
Non-Degreed Mother	0.016	0.014	0.020*	0.021*	0.020*
	(0.009)	(0.009)	(0.009)	(0.009)	(0.009)
Maternal Age at Birth	0.0007	0.0006	0.0004	0.0006	0.0007
	(0.0007)	(0.0007)	(0.0007)	(0.0007)	(0.0007)
Observations	1,974	1,974	1,974	1,974	1,974
R^2	0.05960	0.05958	0.06023	0.05938	0.05781
Adjusted R^2	0.05673	0.05671	0.05736	0.05651	0.05446

* p<0.05, ** p<0.01, *** p<0.001

Results above reflect the final selected life course models after dropping non-significant predictors in the initial step. After removing all non-significant models, we find a sensitive period of age 9 for both pooled- and poverty- related ACEs, as well as a sensitive age 3 period for instability- and deprivation-related ACEs. Because no significant life course models were found for Maltreatment-related ACEs, we report the full results incorporating both initially identified life course models.

Table S4: Coefficient Estimates of Fertility and Health Outcomes (Full Model)

	**BMI z-score**		**Depression**		**Anxiety**		**NEB**		**No. Pregnancies**	
	OLS	OLS	Logit	Logit	Logit	Logit	OLS	OLS	OLS	OLS
	(β)	(β)	$exp(\beta)$	$exp(\beta)$	$exp(\beta)$	$exp(\beta)$	(β)	(β)	(β)	(β)
Constant	0.21	0.26	2.20	2.20	0.00**	0.00**	-1.10*	-1.00	-1.20	-1.10
	(0.45)	(0.46)	(2.20)	(2.20)	(2.80)	(2.80)	(0.63)	(0.63)	(0.88)	(0.88)
**Epigenetic Clock**										
DunedinPACE	$\mathbf{0.29}^{***}$	$\mathbf{0.29}^{***}$	$\mathbf{1.62}^{*}$	$\mathbf{1.62}^{*}$	0.90	0.90	$\mathbf{0.22}^{**}$	$\mathbf{0.21}^{**}$	$\mathbf{0.23}^{*}$	$\mathbf{0.22}^{*}$
	(0.06)	(0.06)	(0.27)	(0.27)	(0.36)	(0.36)	(0.08)	(0.08)	(0.11)	(0.11)
**Life Course Models**										
Sensitive Period (Age 3)		-0.02		1.03		1.03		0.01		0.03
		(0.01)		(0.06)		(0.07)		(0.02)		(0.02)
Sensitive Period (Age 9)		0.03**		1.07		1.04		0.03*		0.04*
		(0.01)		(0.06)		(0.08)		(0.02)		(0.02)
**Covariates**										
Male	-0.10***	-0.10***	0.55***	0.55***	0.70**	0.70**	-0.07*	-0.07*	-0.13**	-0.13**
	(0.02)	(0.02)	(0.10)	(0.10)	(0.13)	(0.13)	(0.03)	(0.03)	(0.04)	(0.04)
Age	0.00	0.00	1.02	1.01	1.31*	1.31*	0.06*	0.05	0.07*	0.07*
	(0.02)	(0.02)	(0.10)	(0.10)	(0.12)	(0.12)	(0.03)	(0.03)	(0.04)	(0.04)
Attended College	-0.04*	-0.04	1.40***	1.45***	1.20	1.22	-0.22***	-0.21***	-0.37***	-0.35***
	(0.02)	(0.02)	(0.10)	(0.10)	(0.13)	(0.14)	(0.03)	(0.03)	(0.04)	(0.04)
Household Income	-0.00	-0.00	1.00	1.00	1.00	1.00	-0.00**	-0.00**	-0.00***	-0.00***
	(0.00)	(0.00)	(0.00)	(0.00)	(0.00)	(0.00)	(0.00)	(0.00)	(0.00)	(0.00)
Parental Household Income	-0.00***	-0.00***	1.00	1.00	1.00	1.00	-0.00*	-0.00	-0.00	-0.00
	(0.00)	(0.00)	(0.00)	(0.00)	(0.00)	(0.00)	(0.00)	(0.00)	(0.00)	(0.00)
White	-0.00	-0.00	1.27	1.30*	1.94***	1.97***	-0.04	-0.03	-0.11*	-0.09
	(0.03)	(0.03)	(0.13)	(0.13)	(0.16)	(0.16)	(0.04)	(0.04)	(0.05)	(0.05)
Weight (Wave 1)	0.01***	0.01***								
	(0.00)	(0.00)								
Observations	1,949	1,949	1,949	1,949	1,949	1,949	1,949	1,949	1,949	1,949
Squared Correlation	0.06	0.06	0.03	0.04	0.02	0.02	0.07	0.07	0.08	0.09

* p<0.05, ** p<0.01, *** p<0.001

Results here reflect the associations between DunedinPACE clock (measured age 15) and subsequent health and life history outcomes (measured age 22). Two models were ran for each outcome of interest: the first model regresses the outcome of interest on the DunedinPACE clock and other control variables; while the second model additionally controls for salient sensitive periods, to determine if epigenetic ageing independently predicts our outcomes of interest. We find that the DunedinPACE clock independently and significantly predicts all outcomes of interest, except for anxiety.

Table S5: Sensitivity Analysis: Life course model selection (Full Model)
Recency as time difference between final exposure and age 15.

	DunedinPACE Ageing				
	Pooled ACEs	Poverty	Instability	Deprivation	Maltreatment
Constant	1.27**	1.27**	1.29**	1.28**	1.28**
	(0.022)	(0.021)	(0.021)	(0.021)	(0.021)
**Life Course Models**					
Sensitive Period (Age 3)	-0.004		**-0.027****	**-0.032***	0.011
	(0.005)		(0.010)	(0.014)	(0.012)
Sensitive Period (Age 9)	**0.012***	**0.021***			0.009
	(0.005)	(0.009)			(0.011)
**Covariates**					
Male	-0.046**	-0.045**	-0.047**	-0.046**	-0.047**
	(0.008)	(0.008)	(0.008)	(0.008)	(0.008)
White	-0.035	-0.035	-0.041*	-0.037*	-0.038*
	(0.018)	(0.018)	(0.018)	(0.018)	(0.018)
White Mother	-0.048**	-0.047**	-0.045**	-0.049**	-0.047**
	(0.017)	(0.017)	(0.017)	(0.017)	(0.017)
Non-Degreed Mother	0.017	0.014	0.020*	0.021*	0.020*
	(0.009)	(0.009)	(0.009)	(0.009)	(0.009)
Maternal Age at Birth	0.0007	0.0006	0.0004	0.0006	0.0007
	(0.0007)	(0.0007)	(0.0007)	(0.0007)	(0.0007)
Observations	1,974	1,974	1,974	1,974	1,974
R^2	0.05988	0.05958	0.06023	0.05938	0.05781
Adjusted R^2	0.05653	0.05671	0.05736	0.05651	0.05446

* p<0.05, ** p<0.01, *** p<0.001

To distinguish between a *sensitive age 9* model and a *recency* model, we re-operationalised recency in terms of the time difference between *age at latest ACE exposure* and *age 15* (when epigenetic clocks were collected). Because more proximate exposures correspond to smaller time gaps, we would expect this measure to be inversely related to one's pace of epigenetic ageing, if more recent adversities indeed do predict a hastened pace of ageing. Results do not differ from our main analyses – adversity exposures had more sensitive periods at age 9 for pooled- and poverty-related ACEs; and age 3 for instability- and deprivation-related ACEs.

Table S6: Sensitivity Analysis: Life course model selection (Full Model)
Recency as ACE count at last exposure weighted by time till age 15.

	DunedinPACE Ageing				
	Pooled ACEs	Poverty	Instability	Deprivation	Maltreatment
Constant	1.28**	1.27**	1.29**	1.28**	1.28**
	(0.022)	(0.021)	(0.021)	(0.021)	(0.021)
**Life Course Models**					
Recency	-0.0009				
	(0.0007)				
Sensitive Period (Age 3)			**-0.027****	**-0.032***	0.011
			(0.010)	(0.014)	(0.012)
Sensitive Period (Age 9)	**0.013****	**0.021***			0.009
	(0.005)	(0.009)			(0.011)
**Covariates**					
Male	-0.046**	-0.045**	-0.047**	-0.046**	-0.047**
	(0.008)	(0.008)	(0.008)	(0.008)	(0.008)
White	-0.035	-0.035	-0.041*	-0.037*	-0.038*
	(0.018)	(0.018)	(0.018)	(0.018)	(0.018)
White Mother	-0.049**	-0.047**	-0.045**	-0.049**	-0.047**
	(0.017)	(0.017)	(0.017)	(0.017)	(0.017)
Non-Degreed Mother	0.016	0.014	0.020*	0.021*	0.020*
	(0.009)	(0.009)	(0.009)	(0.009)	(0.009)
Maternal Age at Birth	0.0006	0.0006	0.0004	0.0006	0.0007
	(0.0007)	(0.0007)	(0.0007)	(0.0007)	(0.0007)
Observations	1,974	1,974	1,974	1,974	1,974
R^2	0.06037	0.05958	0.06023	0.05938	0.05781
Adjusted R^2	0.05702	0.05671	0.05736	0.05651	0.05446

* p<0.05, ** p<0.01, *** p<0.001

We adopted a second alternate *recency* measure, in terms of the count of adversities at last exposure, weighted by time until age 15. Similar to the above, we would expect this measure to be inversely related to one's pace of epigenetic ageing, if more recent exposures predict a hastened pace of ageing. Nonetheless, results from this sensitivity analysis were consistent with our main analysis: sensitive periods of age 9 were significant for pooled- and poverty-related ACEs, while a sensitive age 3 period matters more for instability- and deprivation-related ACEs.

Table S7: Sensitivity Analysis: Life course model selection (Full Model)
Sensitive periods for poverty at birth and age 9

	DunedinPACE Ageing		
	Model 1	Model 2	Model 3
Constant	1.272***	1.274***	1.276***
	(0.021)	(0.021)	(0.021)
**Life Course Models**			
Sensitive Period (Birth)	0.010		0.014
	(0.009)		(0.009)
Sensitive Period (Age 9)	0.015†	**0.018***	
	(0.009)	(0.009)	
**Covariates**			
Male	-0.046***	-0.046***	-0.046***
	(0.008)	(0.008)	(0.008)
White	-0.033†	-0.033†	-0.035†
	(0.018)	(0.018)	(0.018)
White Mother	-0.048**	-0.049**	-0.048**
	(0.016)	(0.016)	(0.016)
Non-Degreed Mother	0.013	0.015	0.016†
	(0.009)	(0.009)	(0.009)
Maternal Age at Birth	0.001	0.001	0.001
	(0.001)	(0.001)	(0.001)
Observations	1,974	1,974	1,974
R^2	0.060	0.059	0.058
Adjusted R^2	0.056	0.056	0.055

† p<0.1, * p<0.05, ** p<0.01, *** p<0.001

We tested if epigenetic ageing was reflective of earlier SES conditions by including poverty status at birth as an additional life course model. Our SCLMA procedure identified poverty *at birth* and *age 9* as sensitive periods contributing to later-life epigenetic ageing (Model 1). After sequentially dropping non-significant predictors according to p-value size, results showed that poverty at age 9 remained predictive of subsequent epigenetic ageing (Model 2). To evaluate the counterfactual scenario in which poverty at birth was retained while poverty at age 9 was excluded, we estimated an additional model removing the age 9 sensitive period instead (Model 3). Results indicated that poverty at birth was no longer statistically significant once considered independently, suggesting that the observed association was driven more strongly by poverty exposure at age 9.

Table S8: Sensitivity Analysis: Life course model selection (Full Model)
Adjusted for epithelial cell composition

	DunedinPACE Ageing				
	Pooled ACEs	Poverty	Instability	Deprivation	Maltreatment
Constant	1.24**	1.25**	1.26**	1.25**	1.25**
	(0.017)	(0.017)	(0.017)	(0.017)	(0.017)
**Life Course Models**					
Sensitive Period (Age 3)			-0.015†	-0.014	0.008
			(0.008)	(0.011)	(0.009)
Sensitive Period (Age 9)	**0.007***	0.012†			0.003
	(0.004)	(0.007)			(0.009)
**Covariates**					
Male	-0.050**	-0.050**	-0.051**	-0.050**	-0.051**
	(0.006)	(0.006)	(0.006)	(0.006)	(0.006)
White	-0.029†	-0.029†	-0.033*	-0.030*	-0.031*
	(0.015)	(0.015)	(0.015)	(0.015)	(0.015)
White Mother	-0.034*	-0.034*	-0.033*	-0.035*	-0.034*
	(0.013)	(0.013)	(0.013)	(0.014)	(0.013)
Non-Degreed Mother	0.014†	0.013†	0.017*	0.017*	0.016*
	(0.007)	(0.007)	(0.007)	(0.007)	(0.007)
Maternal Age at Birth	2.29e-5	-4.12e-5	-0.0002	-7.14e-5	-3.09e-5
	(0.0006)	(0.0006)	(0.0006)	(0.0006)	(0.0006)
Epithelial Cell Comp.	0.771**	0.771**	0.771**	0.771**	0.772**
	(0.024)	(0.025)	(0.025)	(0.025)	(0.025)
Observations	1,974	1,974	1,974	1,974	1,974
R^2	0.37416	0.37386	0.37402	0.37339	0.37325
Adjusted R^2	0.37194	0.37163	0.37179	0.37116	0.37070

† p<0.1, * p<0.05, ** p<0.01, *** p<0.001

We additionally controlled for cell proportions in our LARS regression to identify salient life course models that explained the relationship between adversity exposure and epigenetic ageing. Because epigenetic clocks are trained on methylation patterns that are cell-type specific, it is theoretically important to examine differences in epigenetic clocks, net of differences in cell proportion across individuals. After controlling for epithelial cell composition, only a sensitive age 9 period remained predictive of epigenetic ageing for pooled ACEs. Results from this set of analysis, however, has to be interpreted with caution because cell composition may serve as a mediator on the pathway from social adversity to biological ageing. Adjusting for a mediator may then attenuate part of the effect exerted by adversity exposure on ageing. It is also highly likely in our sample of disadvantaged families that cell-type composition and epigenetic ageing may share a common cause (e.g., depression), which means that cell proportion would also serve as a

collider. Adjusting for epithelial cell composition may then introduce collider bias into our estimates.

Table S9: Sensitivity Analysis: Coefficient Estimates of Fertility and Health OutcomesAdjusted for epithelial cell composition

	**BMI z-score**		**Depression**		**Anxiety**		**NEB**		**No. Pregnancies**	
	OLS	OLS	Logit	Logit	Logit	Logit	OLS	OLS	OLS	OLS
	(β)	(β)	$exp(\beta)$	$exp(\beta)$	$exp(\beta)$	$exp(\beta)$	(β)	(β)	(β)	(β)
Constant	-0.39	-0.34	-1.1	-0.95	-3.2	-3.0	-0.63	-0.56	-1.4	-1.2
	(0.48)	(0.48)	(2.3)	(2.3)	(3.1)	(3.1)	(0.63)	(0.63)	(0.94)	(0.94)
**Epigenetic Clock**										
DunedinPACE	**0.52*****	**0.52*****	0.53	0.52	-0.23	-0.25	**0.19****	**0.18***	0.21	0.20
	(0.07)	(0.07)	(0.35)	(0.35)	(0.46)	(0.46)	(0.09)	(0.09)	(0.14)	(0.14)
**Life Course Models**										
Sensitive Period (Age 3)		-0.006		0.05		-0.04		0.0006		0.03
		(0.01)		(0.06)		(0.08)		(0.02)		(0.02)
Sensitive Period (Age 9)		0.03**		0.04		0.10		0.03**		0.05*
		(0.01)		(0.06)		(0.08)		(0.02)		(0.02)
**Covariates**										
Male	-0.10***	-0.10***	-0.71***	-0.70***	-0.53***	-0.53***	-0.08***	-0.08***	-0.13***	-0.12***
	(0.02)	(0.02)	(0.10)	(0.10)	(0.14)	(0.14)	(0.03)	(0.03)	(0.04)	(0.04)
Age	0.03	0.03	0.007	-0.003	0.08	0.07	0.04	0.03	0.08**	0.07*
	(0.02)	(0.02)	(0.10)	(0.10)	(0.14)	(0.14)	(0.03)	(0.03)	(0.04)	(0.04)
Attended College	-0.04*	-0.03	0.30***	0.33***	0.19	0.21	-0.24***	-0.23***	-0.39***	-0.37***
	(0.02)	(0.02)	(0.10)	(0.11)	(0.14)	(0.15)	(0.03)	(0.03)	(0.04)	(0.04)
Household Income	-0.0002	-0.0002	-4×10^{-5}	3.5×10^{-5}	-0.0005	-0.0005	-0.0006***	-0.0006***	-0.001***	-0.0010***
	(0.0002)	(0.0002)	(0.0008)	(0.0008)	(0.001)	(0.001)	(0.0002)	(0.0002)	(0.0003)	(0.0003)
Parental Household Income	-0.0001	-0.0001	-0.0006	-0.0005	0.0009	0.0009	-0.0003**	-0.0003*	-0.0003	-0.0002
	(0.0001)	(0.0001)	(0.0006)	(0.0006)	(0.0007)	(0.0007)	(0.0001)	(0.0001)	(0.0002)	(0.0002)
White	-0.007	-0.004	0.38***	0.40***	0.45***	0.46***	-0.02	-0.02	-0.11*	-0.09
	(0.03)	(0.03)	(0.14)	(0.14)	(0.17)	(0.17)	(0.04)	(0.04)	(0.06)	(0.06)
Epithelial Composition	-0.37***	-0.36***	0.12	0.14	0.24	0.25	0.02	0.03	0.14	0.15
	(0.10)	(0.10)	(0.46)	(0.46)	(0.62)	(0.62)	(0.13)	(0.13)	(0.19)	(0.19)
Observations	1,797	1,797	1,797	1,797	1,797	1,797	1,797	1,797	1,797	1,797
Squared Correlation	0.06080	0.06299	0.04474	0.04561	0.02017	0.02120	0.07838	0.08053	0.08914	0.09254
Pseudo R^2	0.05079	0.05269	0.03395	0.03456	0.02069	0.02174	0.04597	0.04729	0.03607	0.03751
BIC	2,173.8	2,184.6	2,409.7	2,423.2	1,586.9	1,600.3	3,111.2	3,122.0	4,551.1	4,559.4

* p<0.05, ** p<0.01, *** p<0.001

We adjusted for epithelial cell composition to examine if intrinsic epigenetic ageing still predicted later life social- and life history outcomes. After controlling for cell-type composition, the DunedinPACE clock still remained highly predictive of BMI z-scores ($p < 0.001$), but not mental health outcomes. Epigenetic ageing also remained predictive of children ever born (NEB) at the 0.05 significance level. This potentially suggests that epigenetic ageing matters more for physical health outcomes, rather than those of a social or emotional nature.

Table S10: Mediating Effects of Epigenetic Accelerated Ageing (EAA) Adjusted for Epithelial Cell Composition

Effect	Path	β	95% CI: CI Lower	95% CI: CI Upper
**Outcome: BMI z-score**				
Average Mediation Effect	ACE → DunedinPACE → BMI z-score	**0.009***	0.002	0.020
Average Direct Effect	ACE → BMI z-score	**0.020.**	-0.0029	0.040
Total Effect	ACE → BMI z-score	**0.029***	0.004	0.050
Prop. Mediated		0.30		
**Outcome: Depression**				
Average Mediation Effect	ACE → DunedinPACE → Depression	0.001	-0.002	0.00
Average Direct Effect	ACE → Depression	0.018	-0.009	0.04
Total Effect	ACE → Depression	0.018	-0.009	0.04
Prop. Mediated		0.029		
**Outcome: Anxiety**				
Average Mediation Effect	ACE → DunedinPACE → Anxiety	-0.1e-03	-0.002	0.00
Average Direct Effect	ACE → Anxiety	0.007	-0.013	0.03
Total Effect	ACE → Anxiety	0.006	-0.014	0.02
Prop. Mediated		0.028		
**Outcome: No. of Pregnancies**				
Average Mediation Effect	ACE → DunedinPACE → Pregnancies	**0.003.**	-0.5e-03	0.01
Average Direct Effect	ACE → Pregnancies	**0.051***	0.007	0.10
Total Effect	ACE → Pregnancies	**0.054***	0.010	0.10
Prop. Mediated		0.059		
**Outcome: NEB**				
Average Mediation Effect	ACE → DunedinPACE → NEB	**2.66e-03***	1.54e-05	0.01
Average Direct Effect	ACE → NEB	**3.58e-02***	4.53e-03	0.07
Total Effect	ACE → NEB	**3.84e-02***	6.98e-03	0.07
Prop. Mediated		0.070		

· p<0.1, * p<0.05, ** p<0.01, *** p<0.001

In investigating if epigenetic ageing mediated the relationship between adversity exposure and health/life history outcomes, we additionally adjusted for epithelial cell composition as sensitivity analysis. Results did not differ much from our original analysis – the DunedinPACE clock remained a significant mediator for BMI and the number of children ever born (NEB). Significance was however attenuated for number of pregnancies. In line with earlier analysis, the DunedinPACE clock serves as a much stronger mediator for BMI z-scores (proportion mediated = 0.3) compared to NEB (proportion mediated = 0.07). This aligns with our original conclusions that epigenetic ageing plays a stronger role in physical- rather than social or emotional outcomes.

Table S11: Mediating Effects of Epithelial Cell Composition
Epithelial Cell Composition and DunedinPACE clock collected in Wave 6.

**Adversity: Pooled**				
Effect	Path	β	CI Lower	CI Upper
Average Mediation Effect	Pooled ACEs → Epithelial Comp. → DunedinPACE	0.002	-0.005	0.010
Average Direct Effect	Pooled ACEs → DunedinPACE	0.008**	0.003	0.01
Total Effect	Pooled ACEs → DunedinPACE	0.010*	0.002	0.020
Prop. Mediated		0.18		
**Adversity: Poverty**				
Effect	Path	β	CI Lower	CI Upper
Average Mediation Effect	Poverty → Epithelial Comp. → DunedinPACE	0.003	-0.013	0.020
Average Direct Effect	Poverty → DunedinPACE	0.012***	-0.017	0.007
Total Effect	Poverty → DunedinPACE	0.020*	0.002	0.040
Prop. Mediated		0.17		
**Adversity: Instability**				
Effect	Path	β	CI Lower	CI Upper
Average Mediation Effect	Instability → Epithelial Comp. → DunedinPACE	**-0.020***	-0.035	0.001
Average Direct Effect	Instability → DunedinPACE	-0.006	-0.019	0.01
Total Effect	Instability → DunedinPACE	-0.026*	-0.045	0.01
Prop. Mediated		0.76		
**Adversity: Deprivation**				
Effect	Path	β	CI Lower	CI Upper
Average Mediation Effect	Deprivation → Epithelial Comp. → DunedinPACE	**-0.025***	-0.044	-0.00
Average Direct Effect	Deprivation → DunedinPACE	-0.004	-0.022	0.01
Total Effect	Deprivation → DunedinPACE	-0.029*	-0.055	-0.00
Prop. Mediated		0.85		

. p<0.1, * p<0.05, ** p<0.01, *** p<0.001

We conducted a follow-up mediation analysis to investigate if epithelial cell composition lies on the path between adversity exposure and epigenetic ageing. If epithelial cell composition is a mediator, then it will explain why some life course models attenuated in significance after adjusting for cell proportions. Of the three adversities that attenuated in significance (*poverty; instability; deprivation*), epithelial cell composition was found to be a significant mediator in two of them (*instability; deprivation*). Epithelial composition mediated about 76% of the total effect of adversity on epigenetic ageing for instability-related ACEs, and 85% of total effects for deprivation-related ACEs.

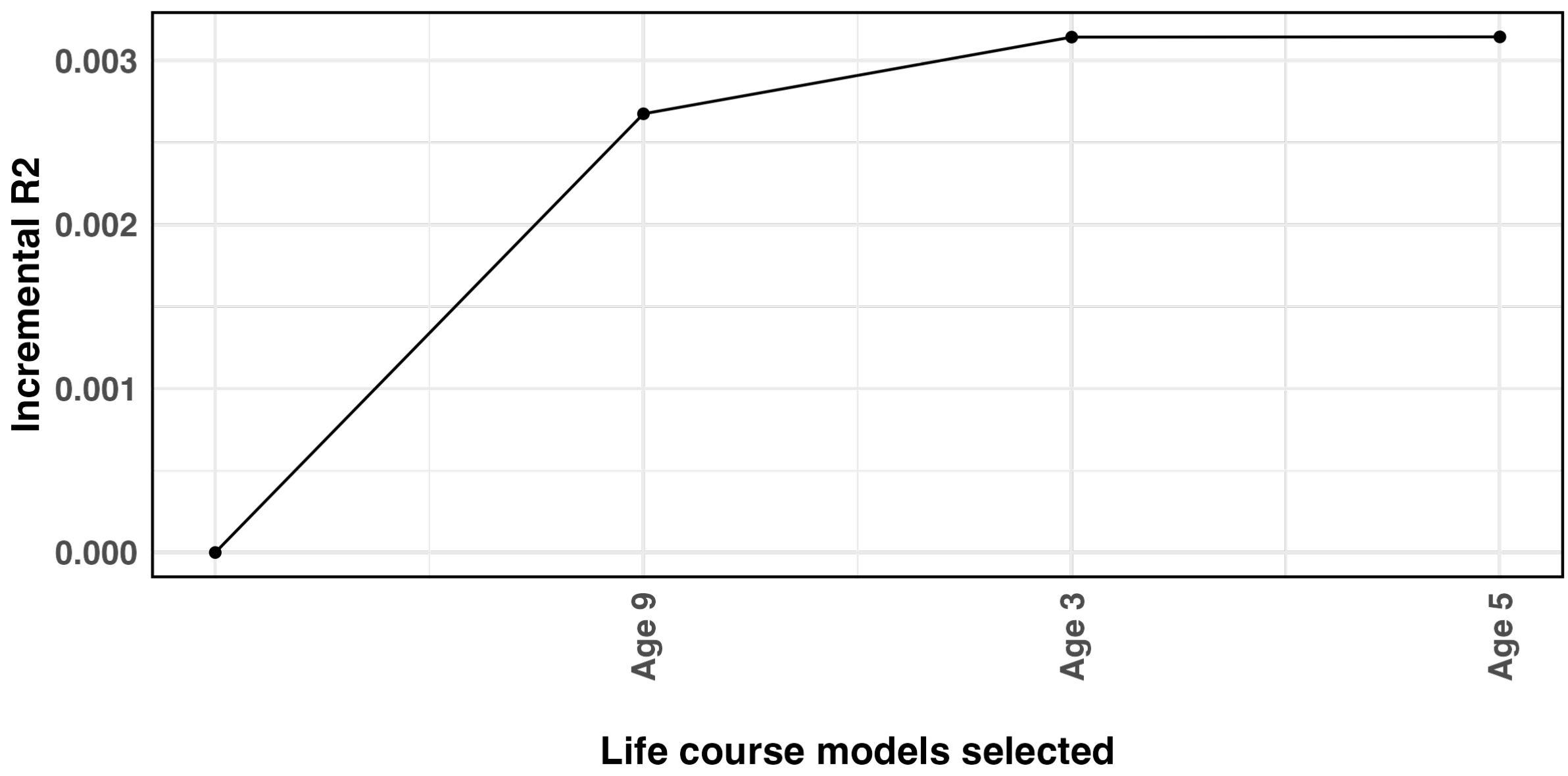

Figure S2: Elbow plot for model selection (Pooled ACEs)

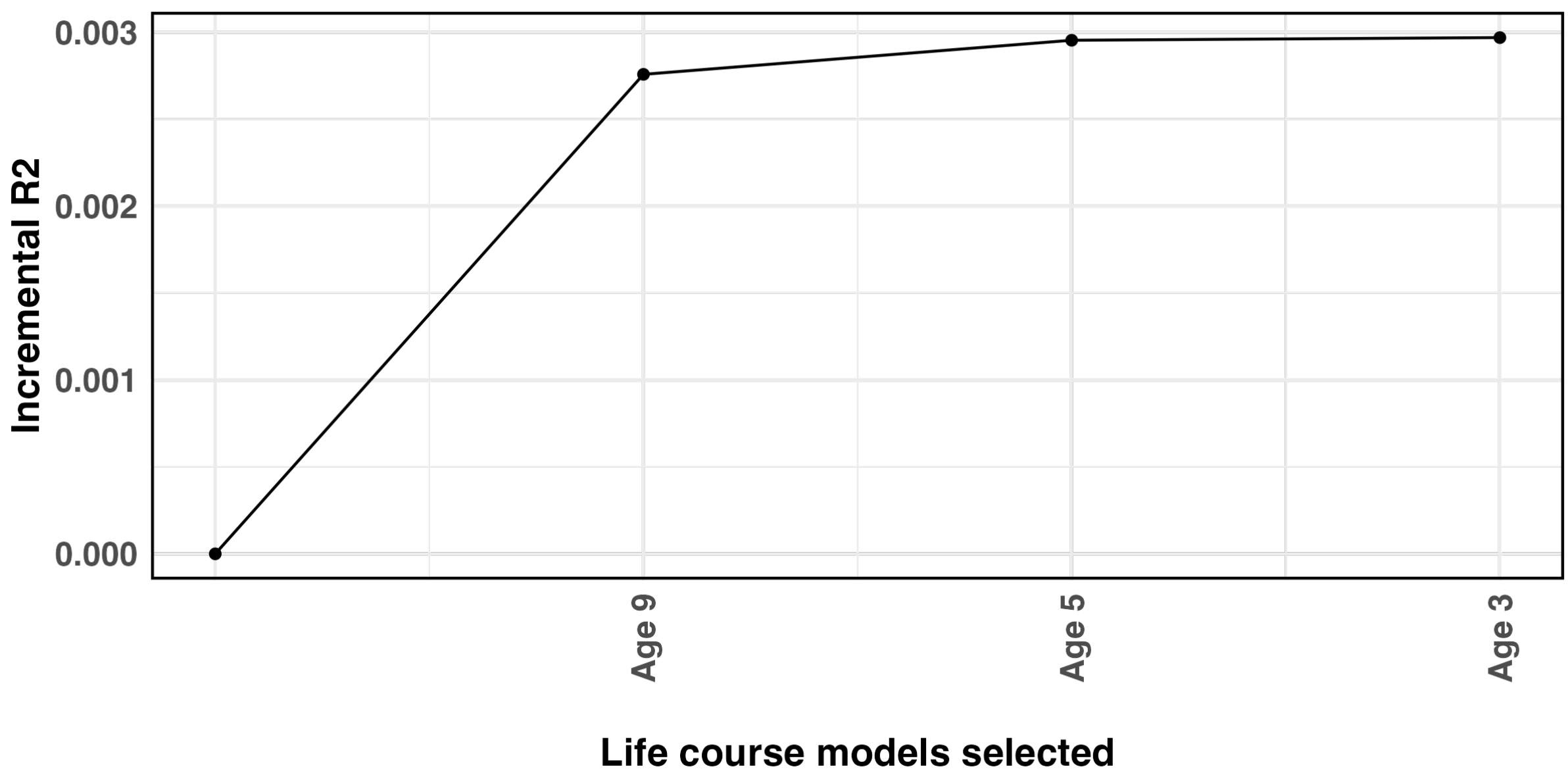

Figure S3: Elbow plot for model selection (Poverty)

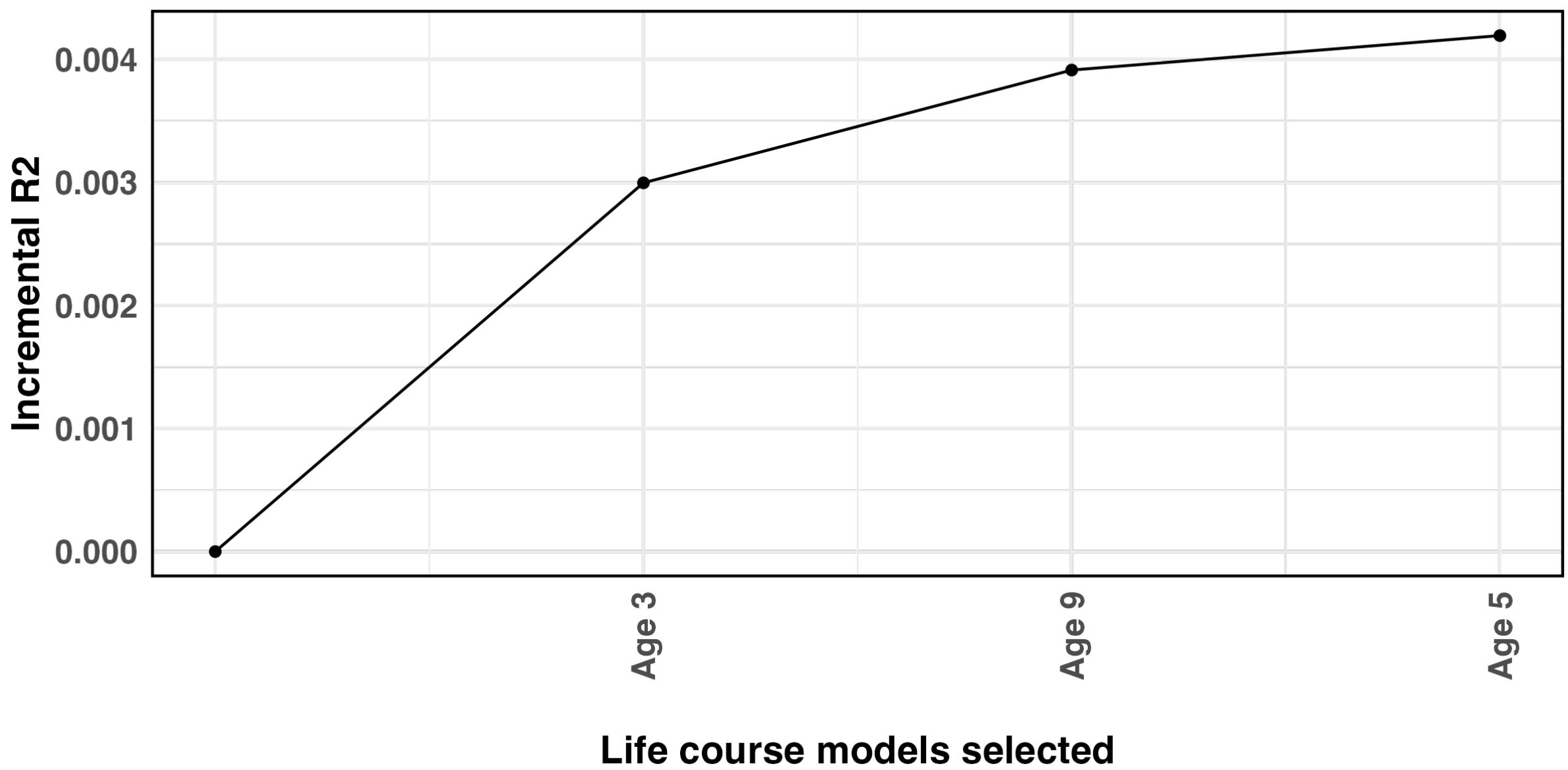

Figure S4: Elbow plot for model selection (Instability)

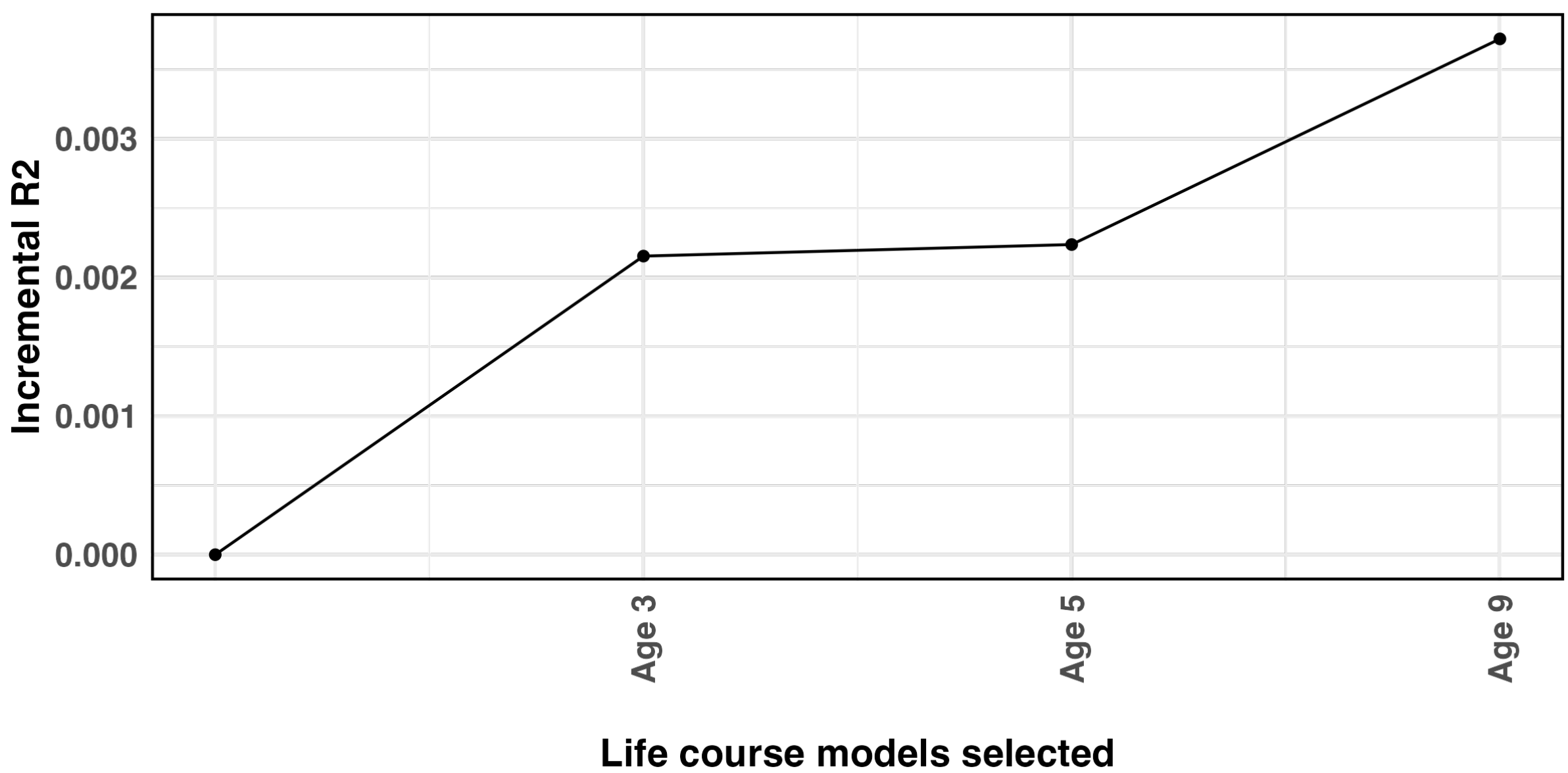

Figure S5: Elbow plot for model selection (Deprivation)

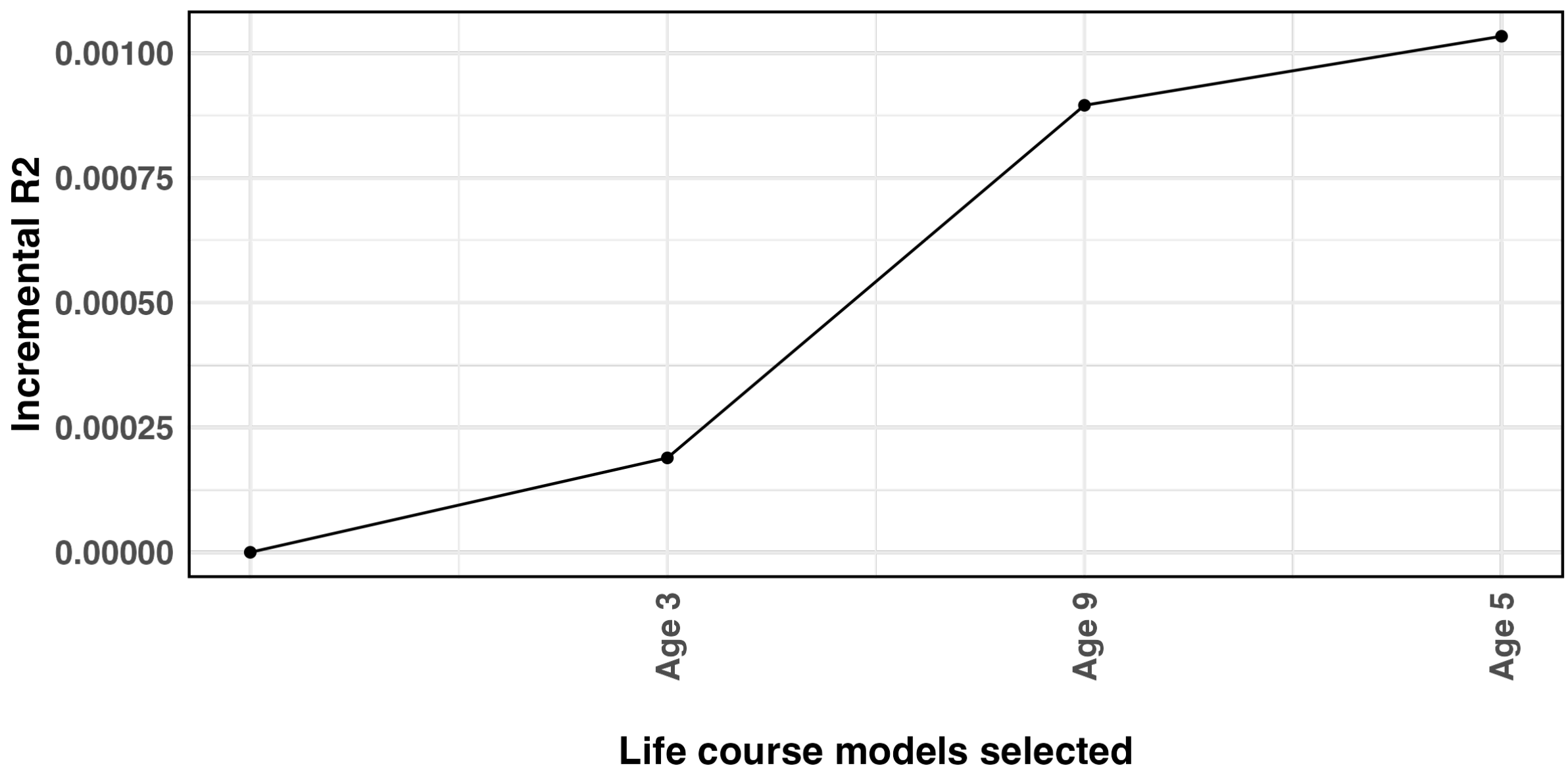

Figure S6: Elbow plot for model selection (Maltreatment)

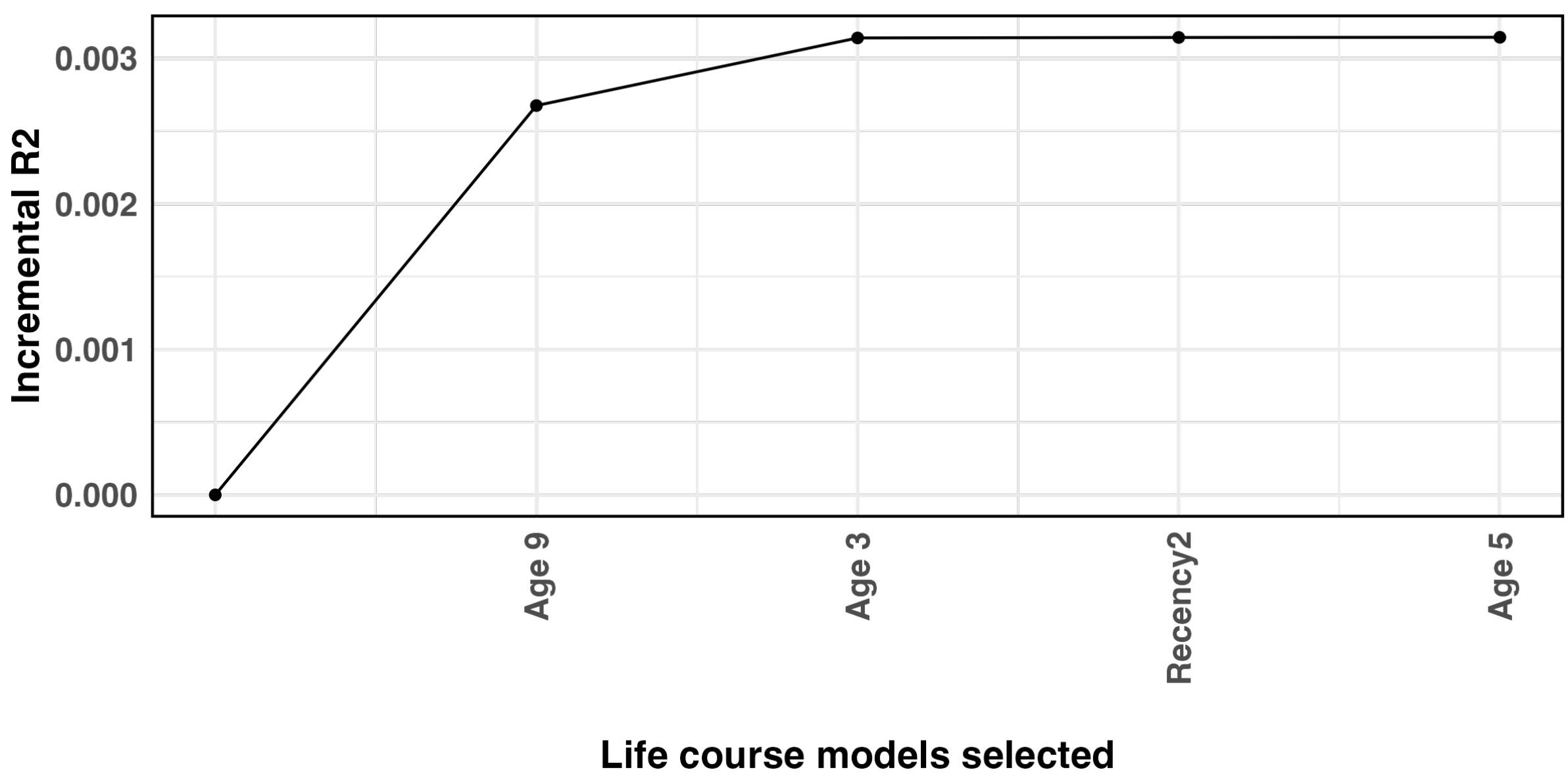

Figure S7: Elbow plot for model selection (Pooled ACEs)
Sensitivity Analysis: *Recency* defined as time at last exposure until age 15.

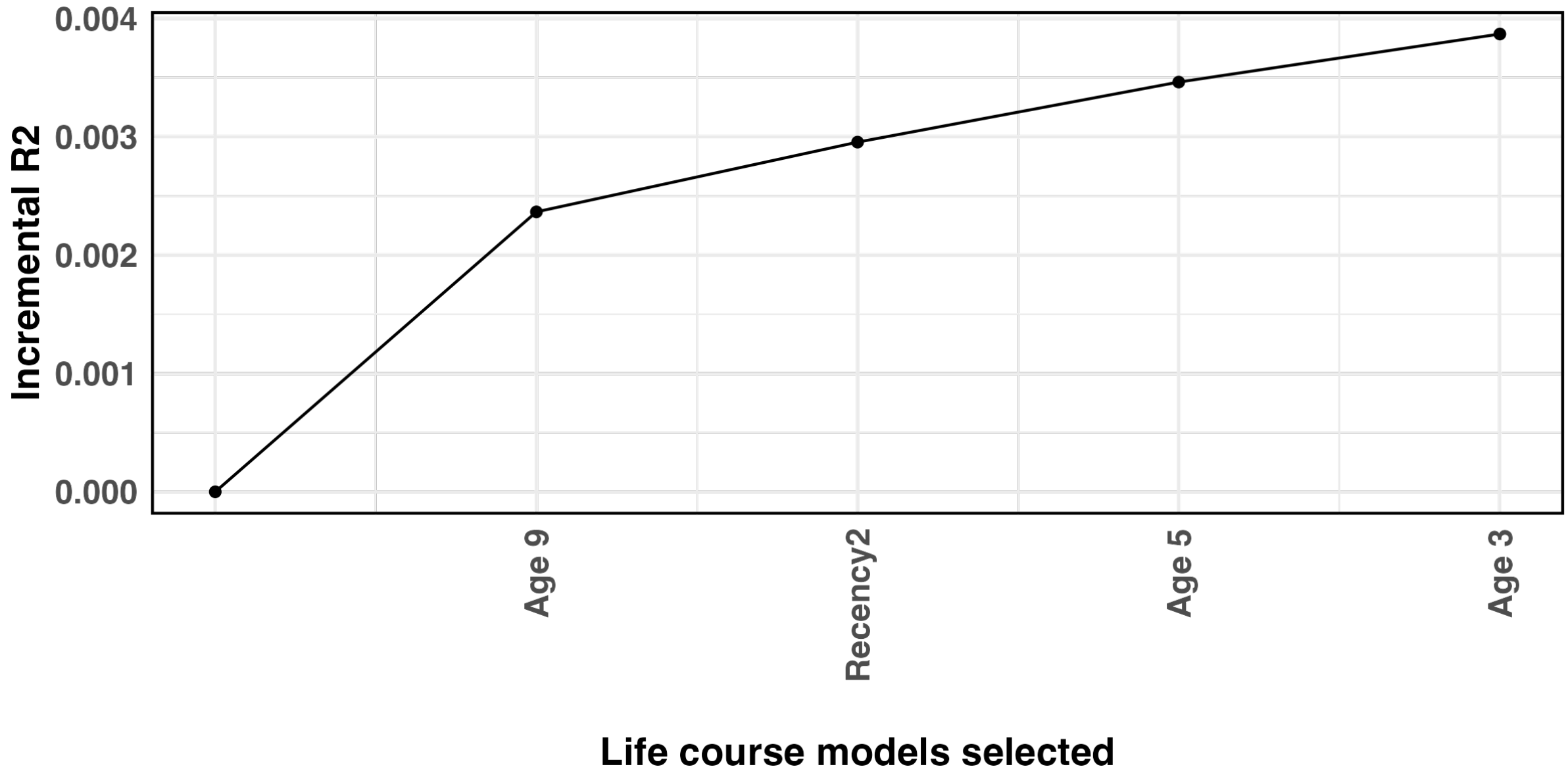

Figure S8: Elbow plot for model selection (Poverty)
Sensitivity Analysis: *Recency* defined as time at last exposure until age 15.

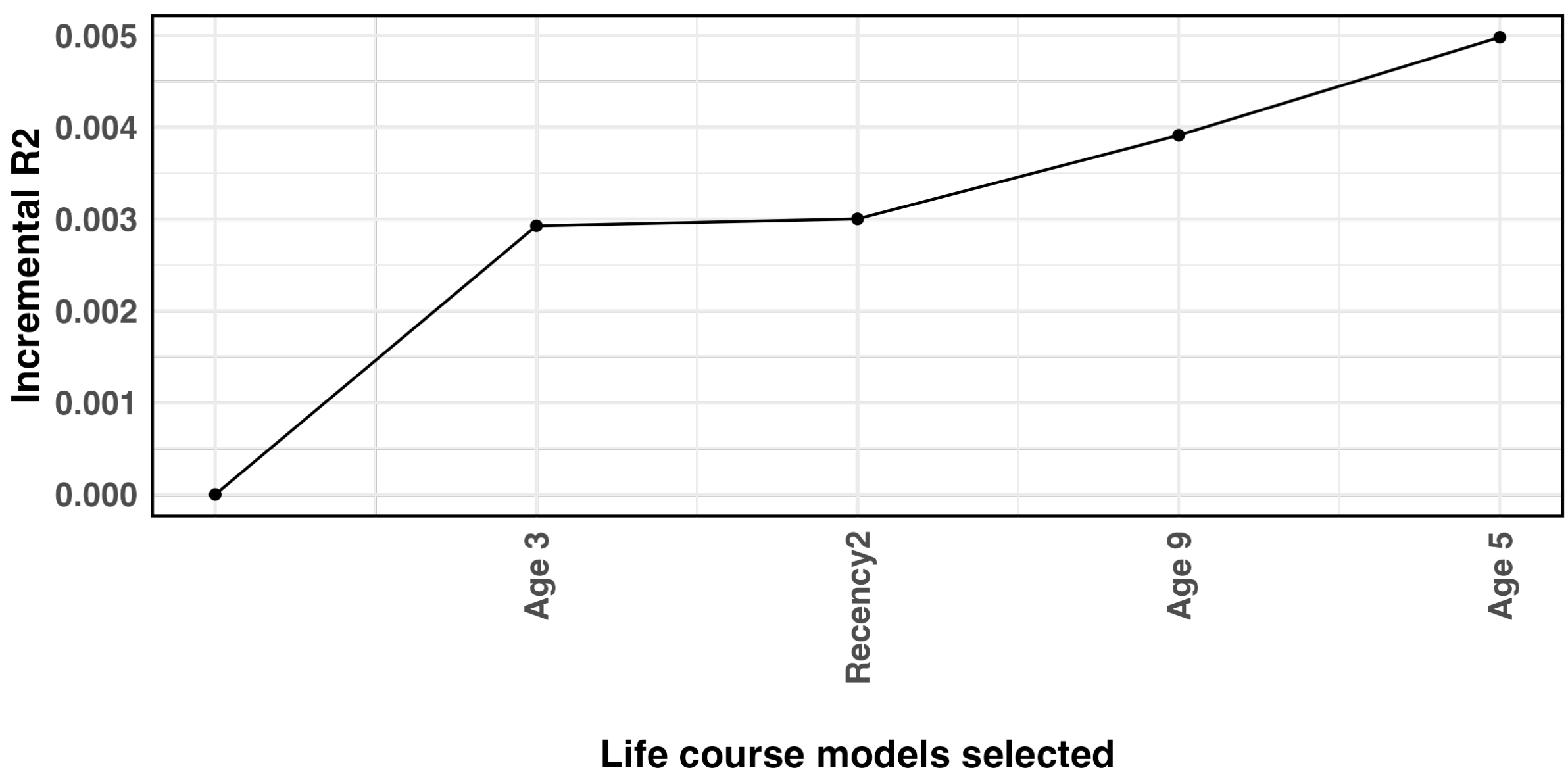

Figure S9: Elbow plot for model selection (Instability)
Sensitivity Analysis: *Recency* defined as time at last exposure until age 15.

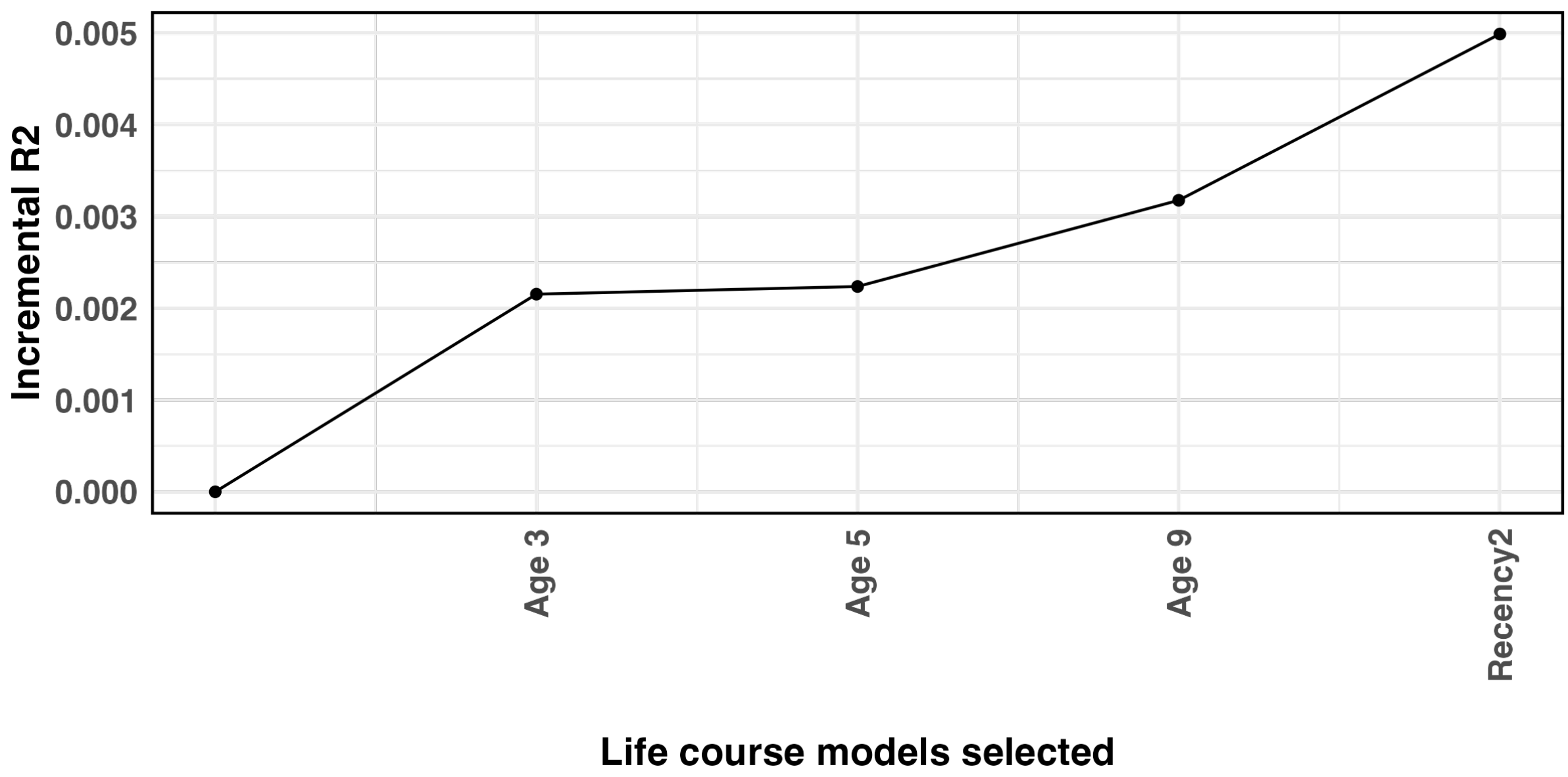

Figure S10: Elbow plot for model selection (Deprivation)
Sensitivity Analysis: *Recency* defined as time at last exposure until age 15.

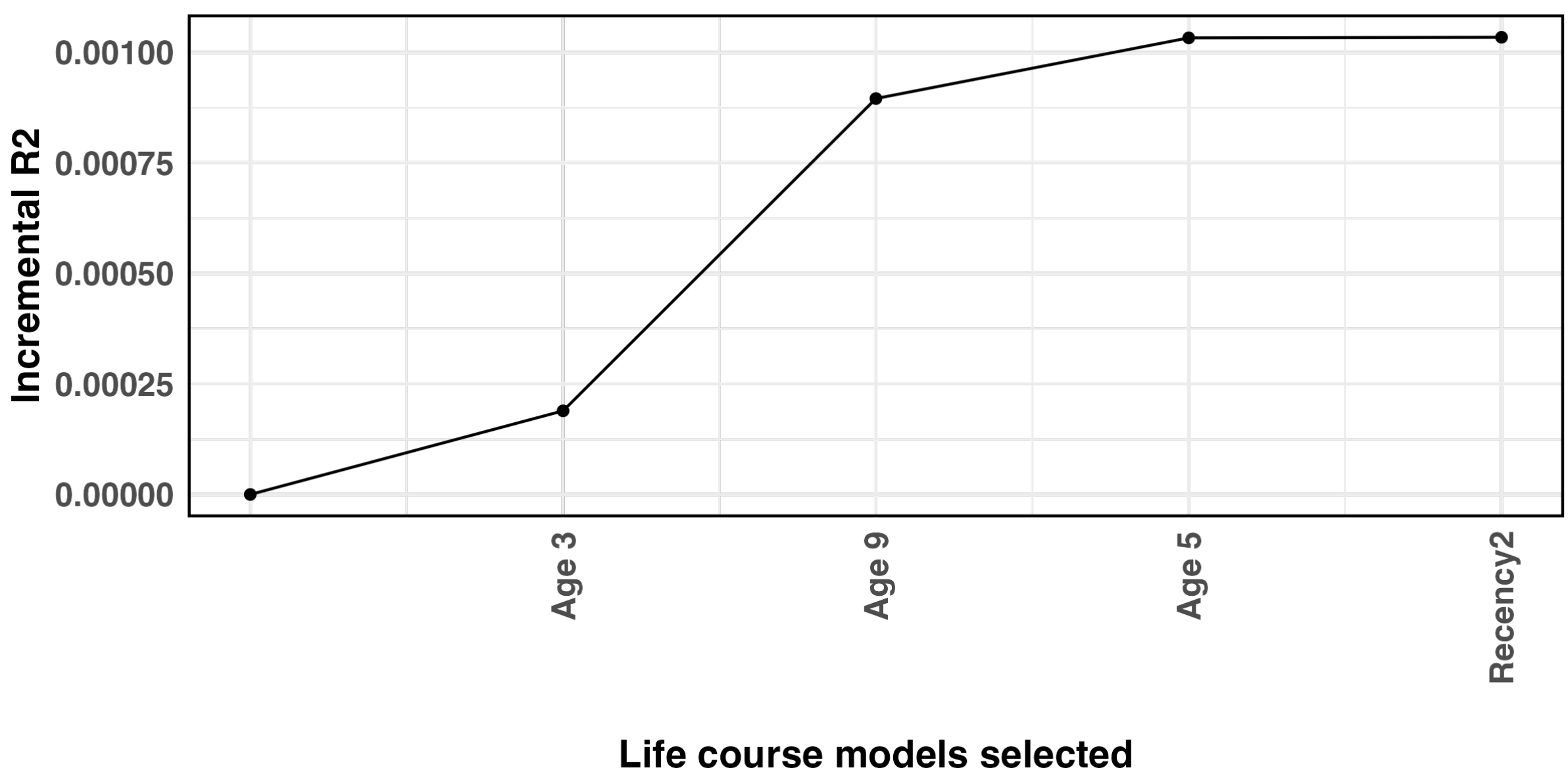

Figure S11: Elbow plot for model selection (Maltreatment)
Sensitivity Analysis: *Recency* defined as time at last exposure until age 15.

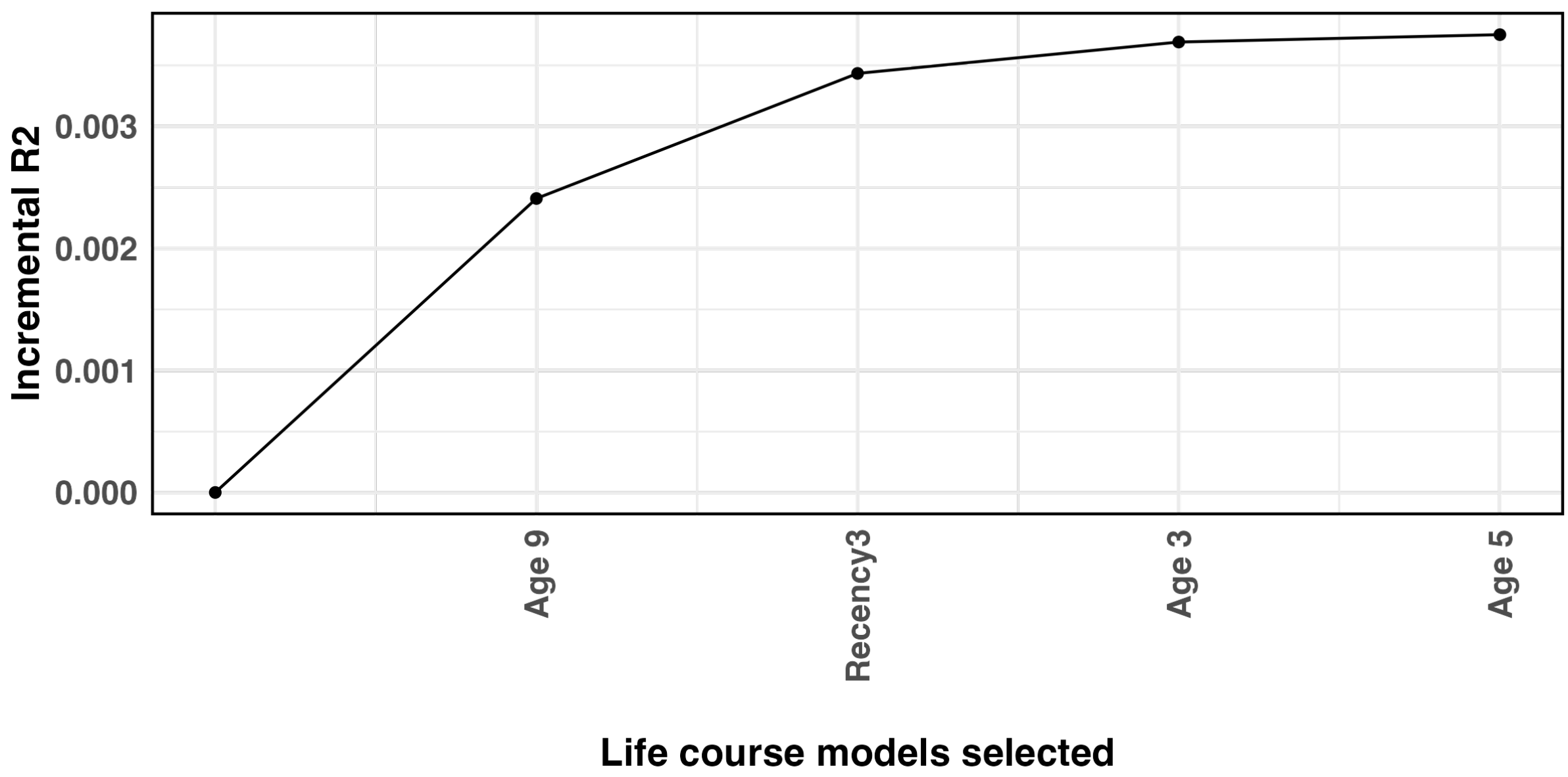

Figure S12: Elbow plot for model selection (Pooled ACEs)
Sensitivity Analysis: *Recency* defined as the count of ACEs at the last exposure, weighted by time until age 15.

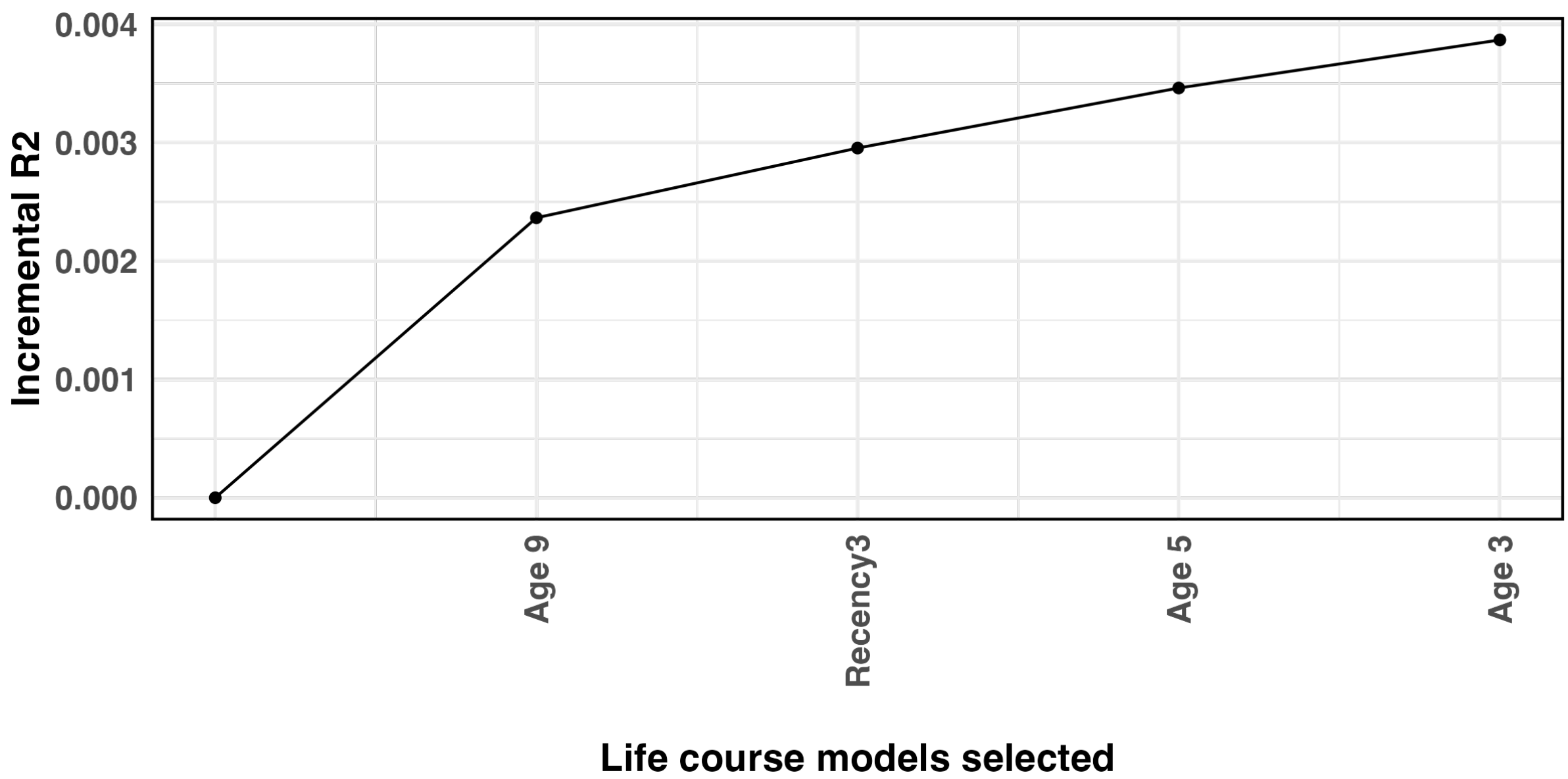

Figure S13: Elbow plot for model selection (Poverty)
Sensitivity Analysis: *Recency* defined as the count of ACEs at the last exposure, weighted by time until age 15.

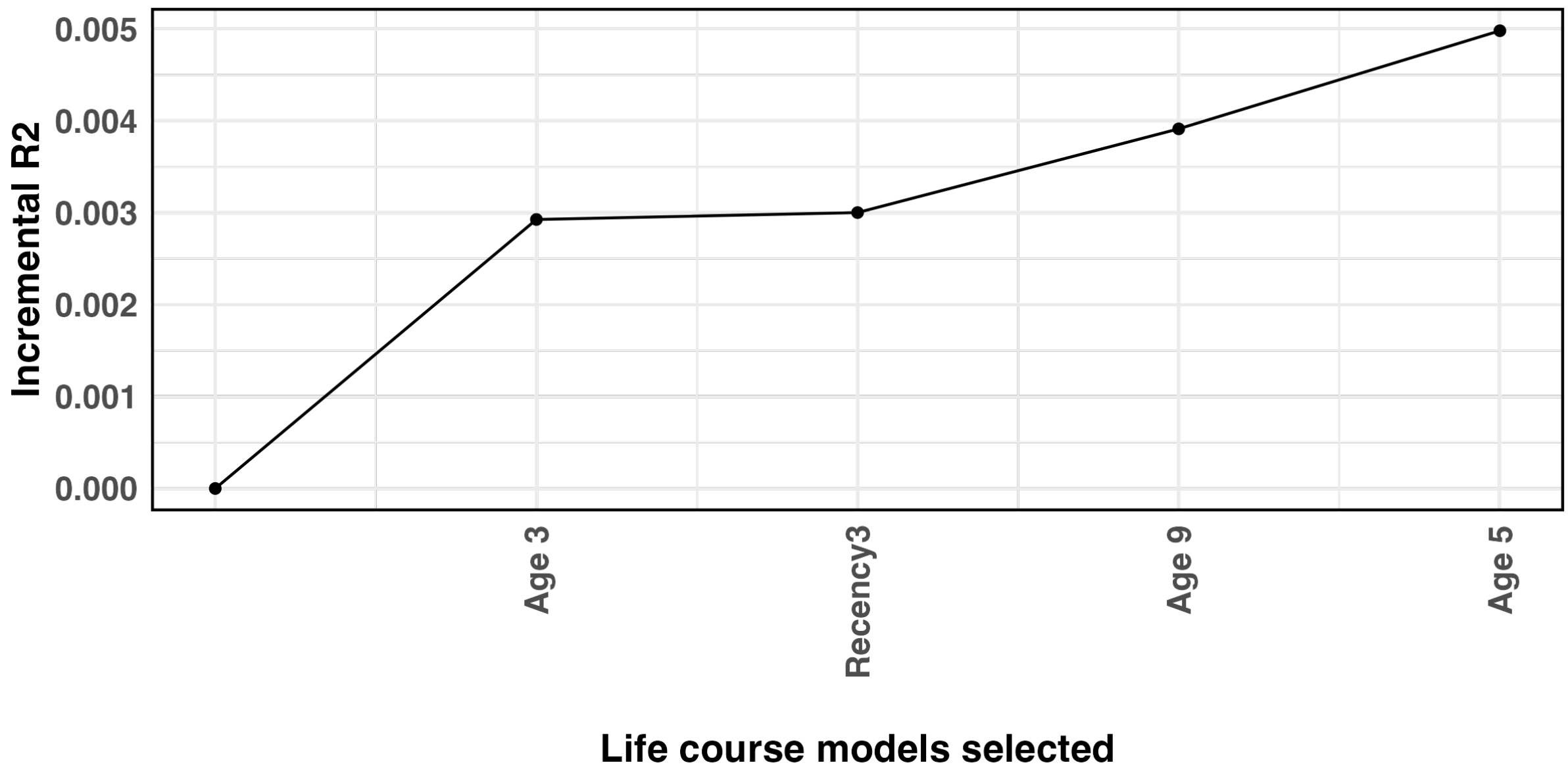

Figure S14: Elbow plot for model selection (Instability)
Sensitivity Analysis: *Recency* defined as the count of ACEs at the last exposure, weighted by time until age 15.

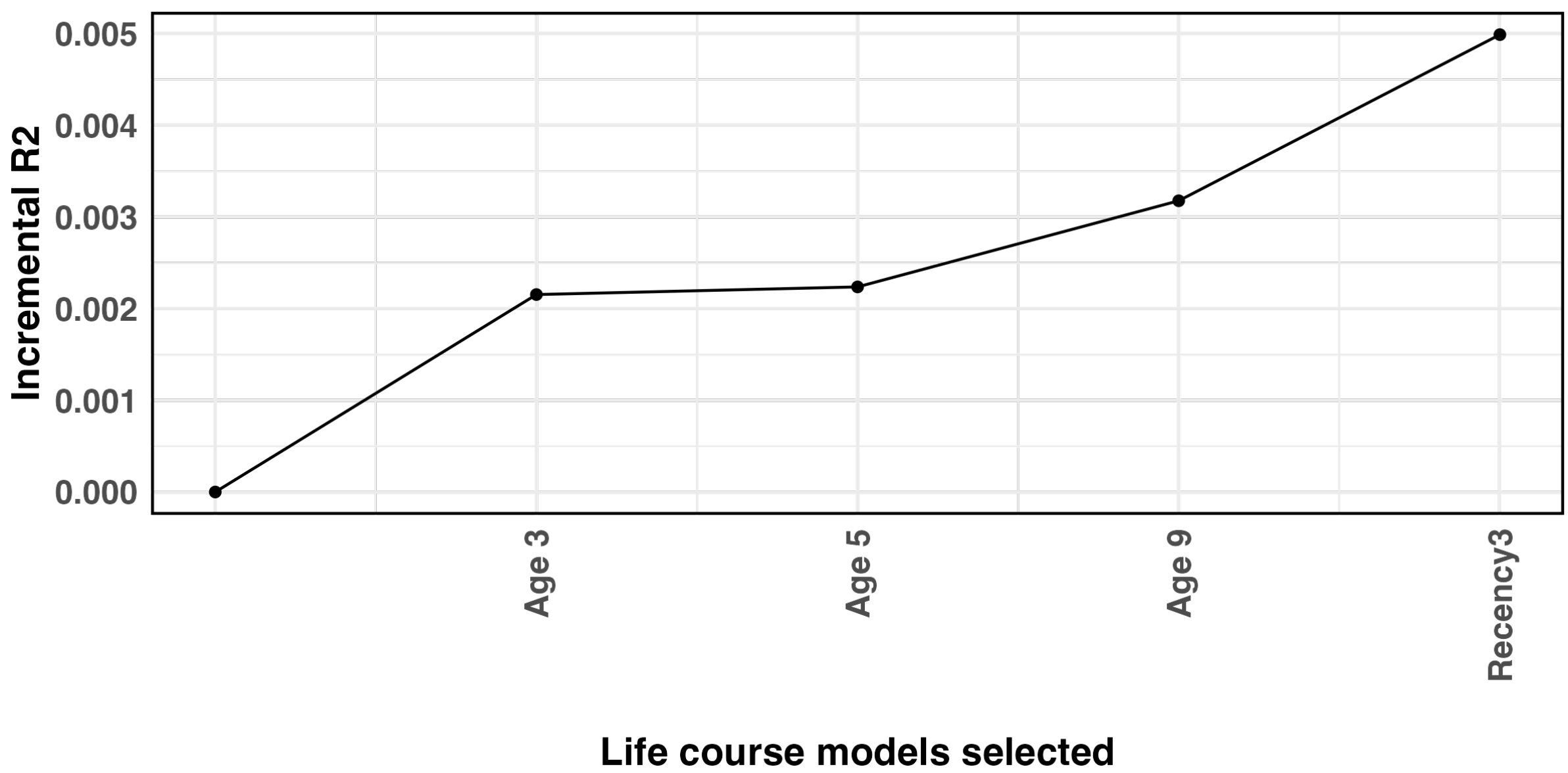

Figure S15: Elbow plot for model selection (Deprivation)
Sensitivity Analysis: *Recency* defined as the count of ACEs at the last exposure, weighted by time until age 15.

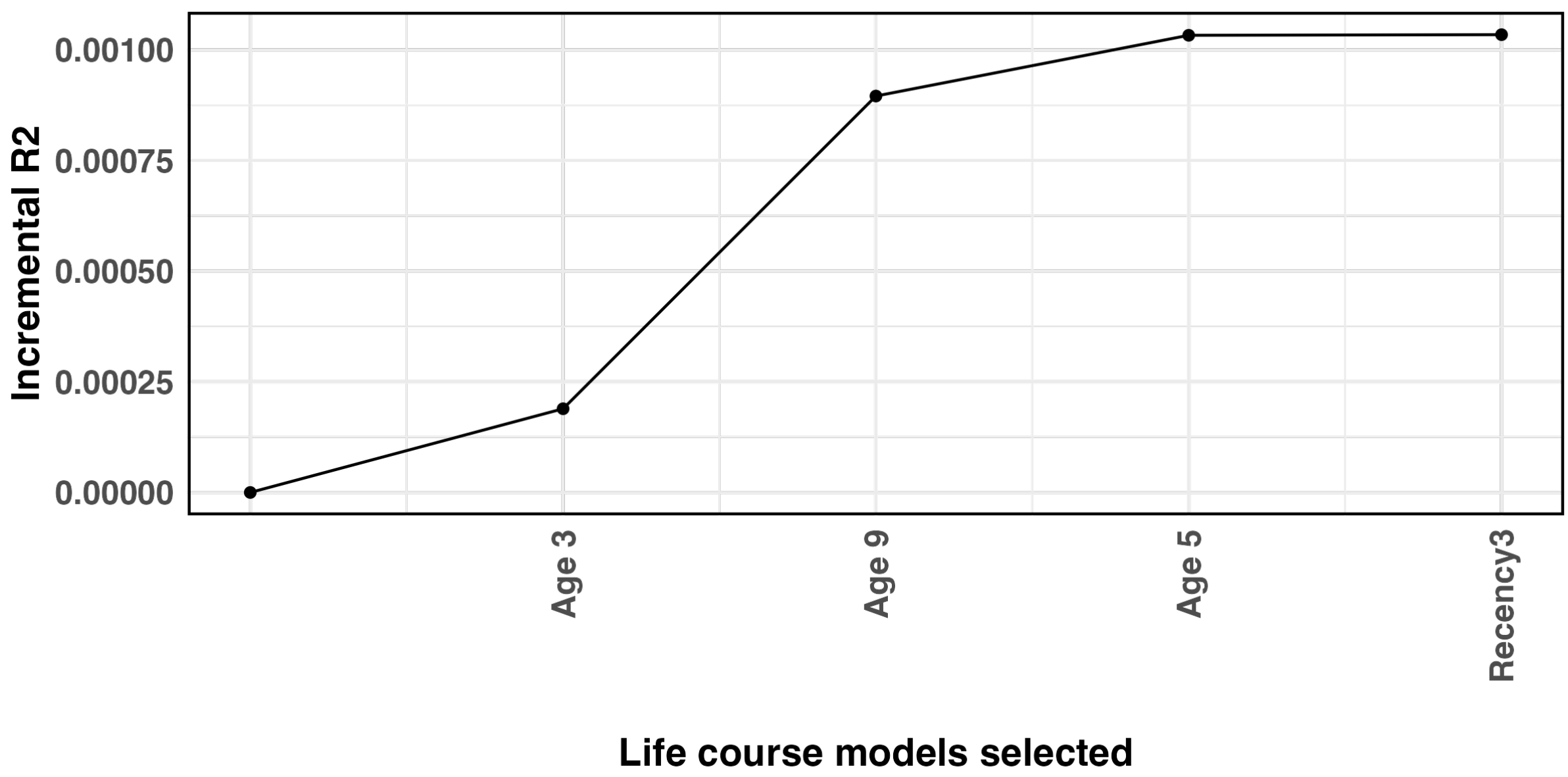

Figure S16: Elbow plot for model selection (Maltreatment)
Sensitivity Analysis: *Recency* defined as the count of ACEs at the last exposure, weighted by time until age 15.

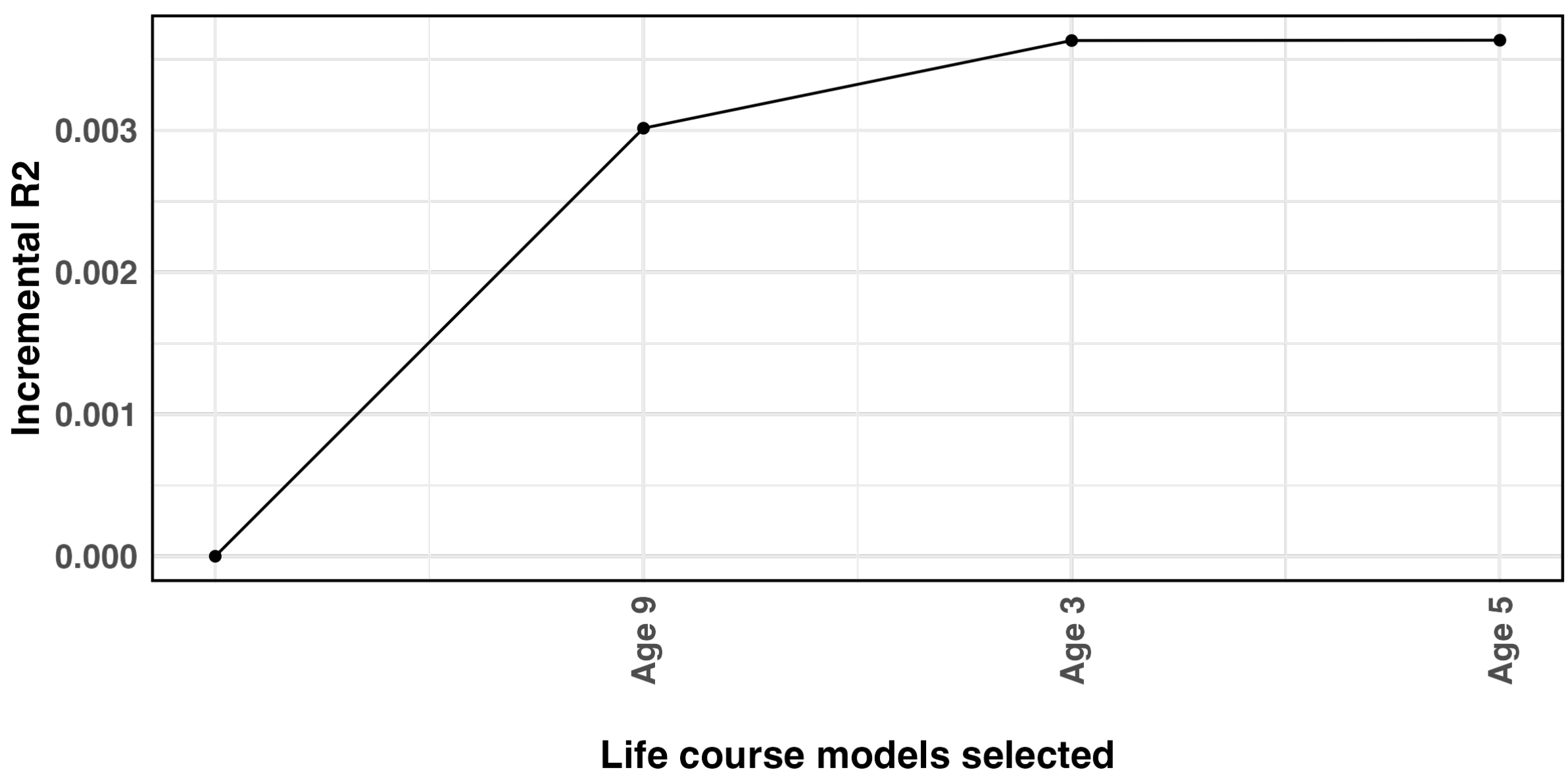

Figure S17: Elbow plot for model selection (Pooled ACEs)
Sensitivity Analysis: *Cumulative Risk score* summed across three timepoints, with high-adversity categories combined.

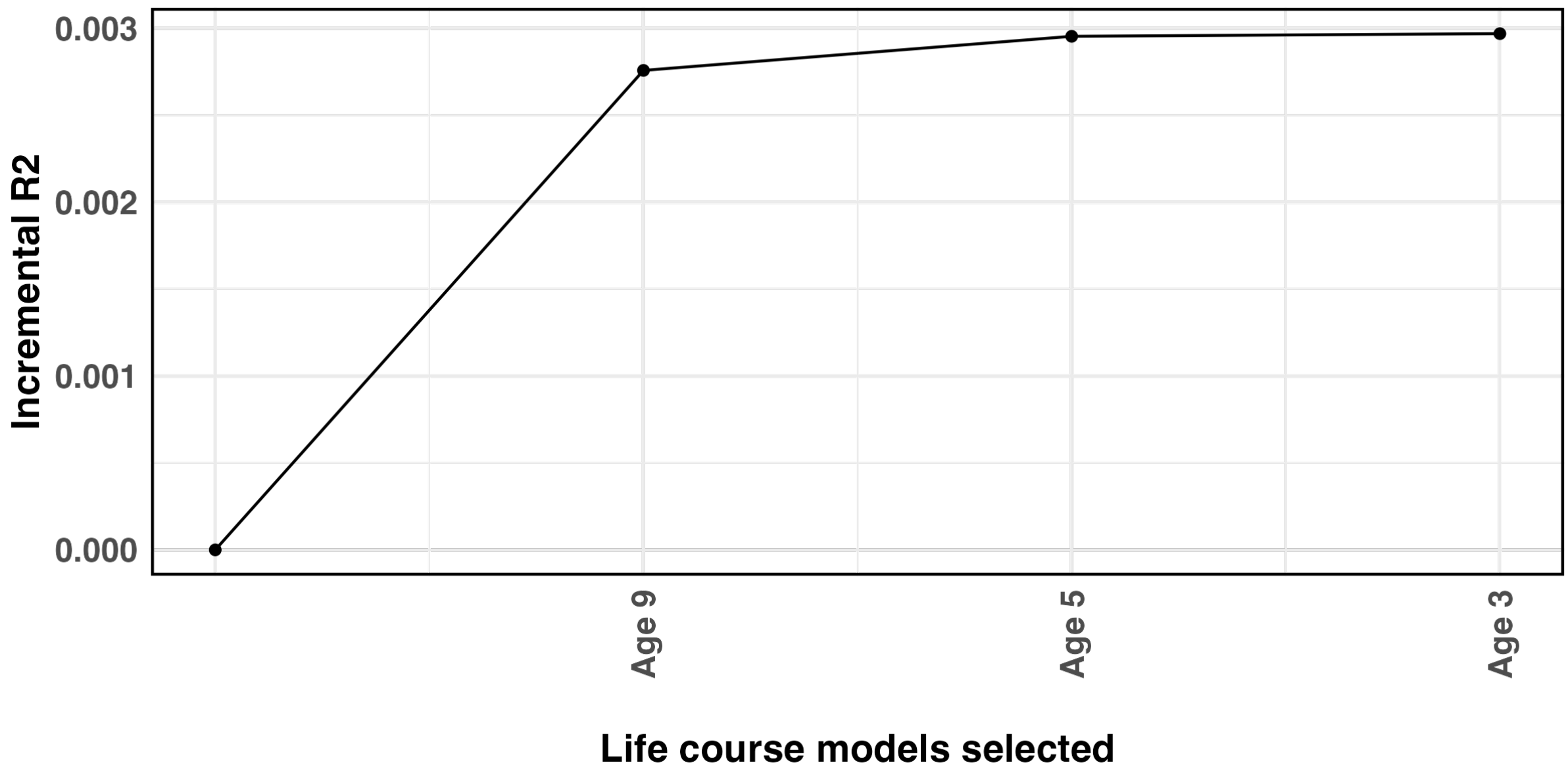

Figure S18: Elbow plot for model selection (Poverty)
Sensitivity Analysis: *Cumulative Risk score* summed across three timepoints, with high-adversity categories combined.

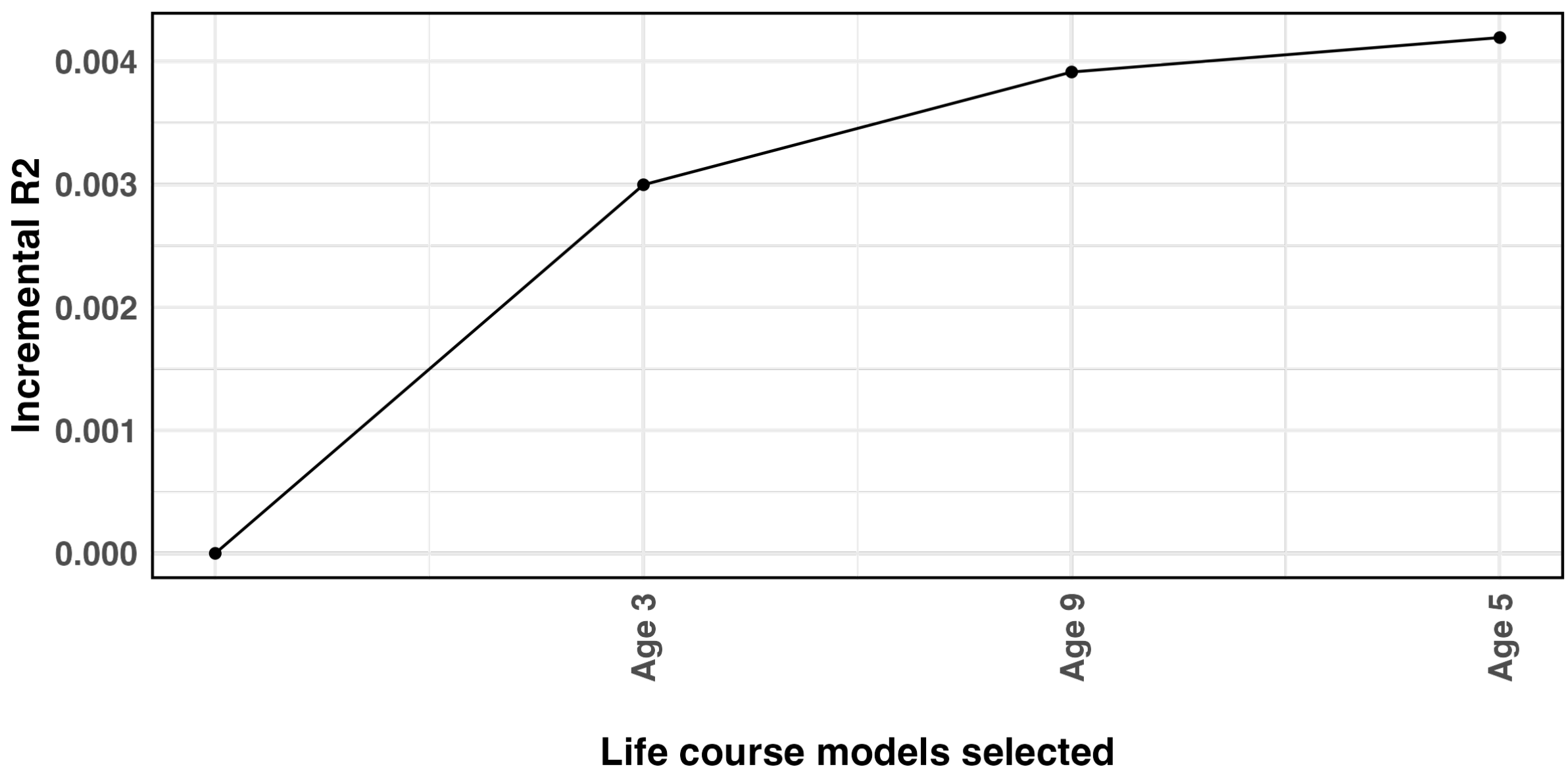

Figure S19: Elbow plot for model selection (Instability)
Sensitivity Analysis: *Cumulative Risk score* summed across three timepoints, with high-adversity categories combined.

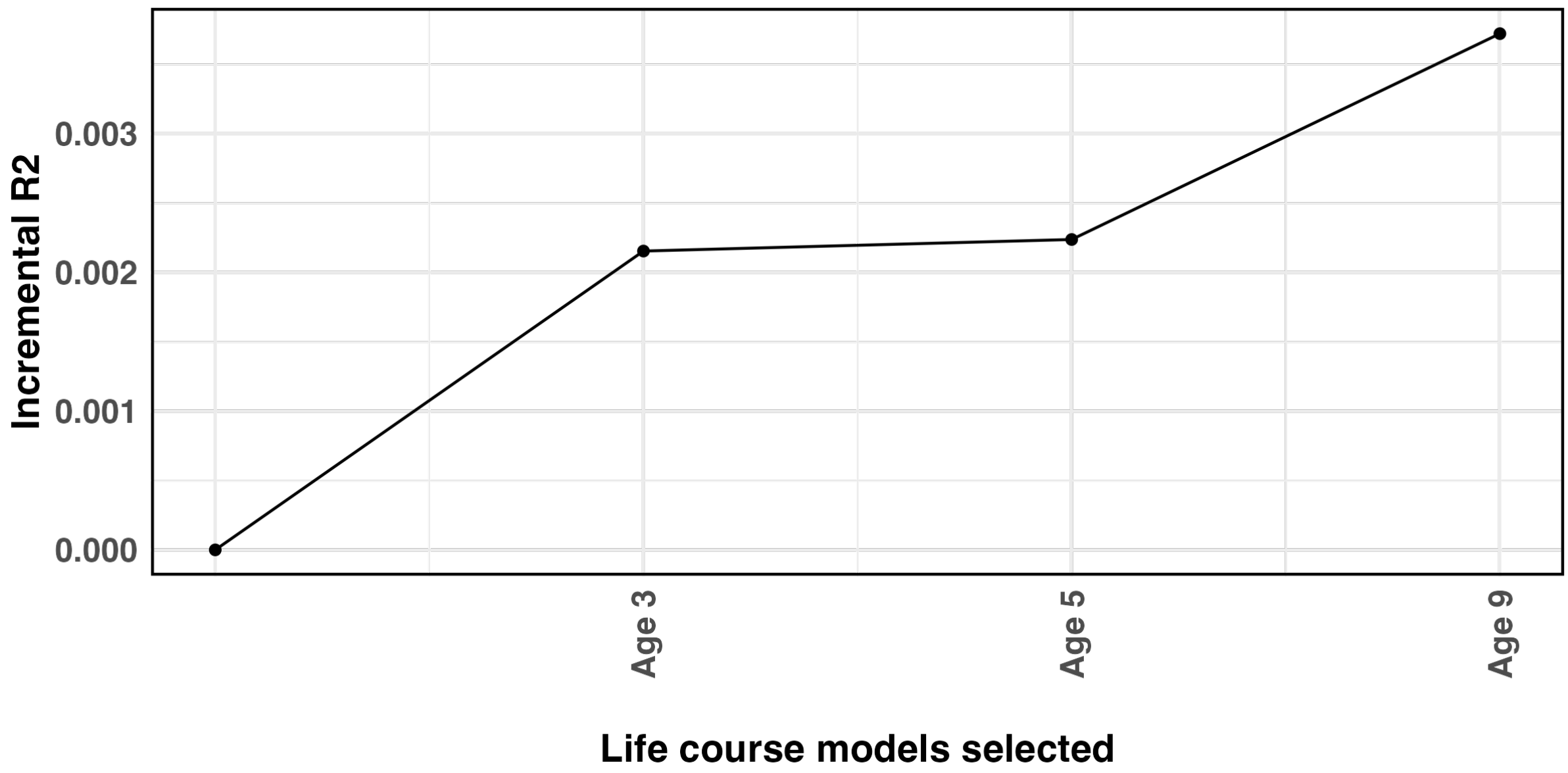

Figure S20: Elbow plot for model selection (Deprivation)
Sensitivity Analysis: *Cumulative Risk score* summed across three timepoints, with high-adversity categories combined.

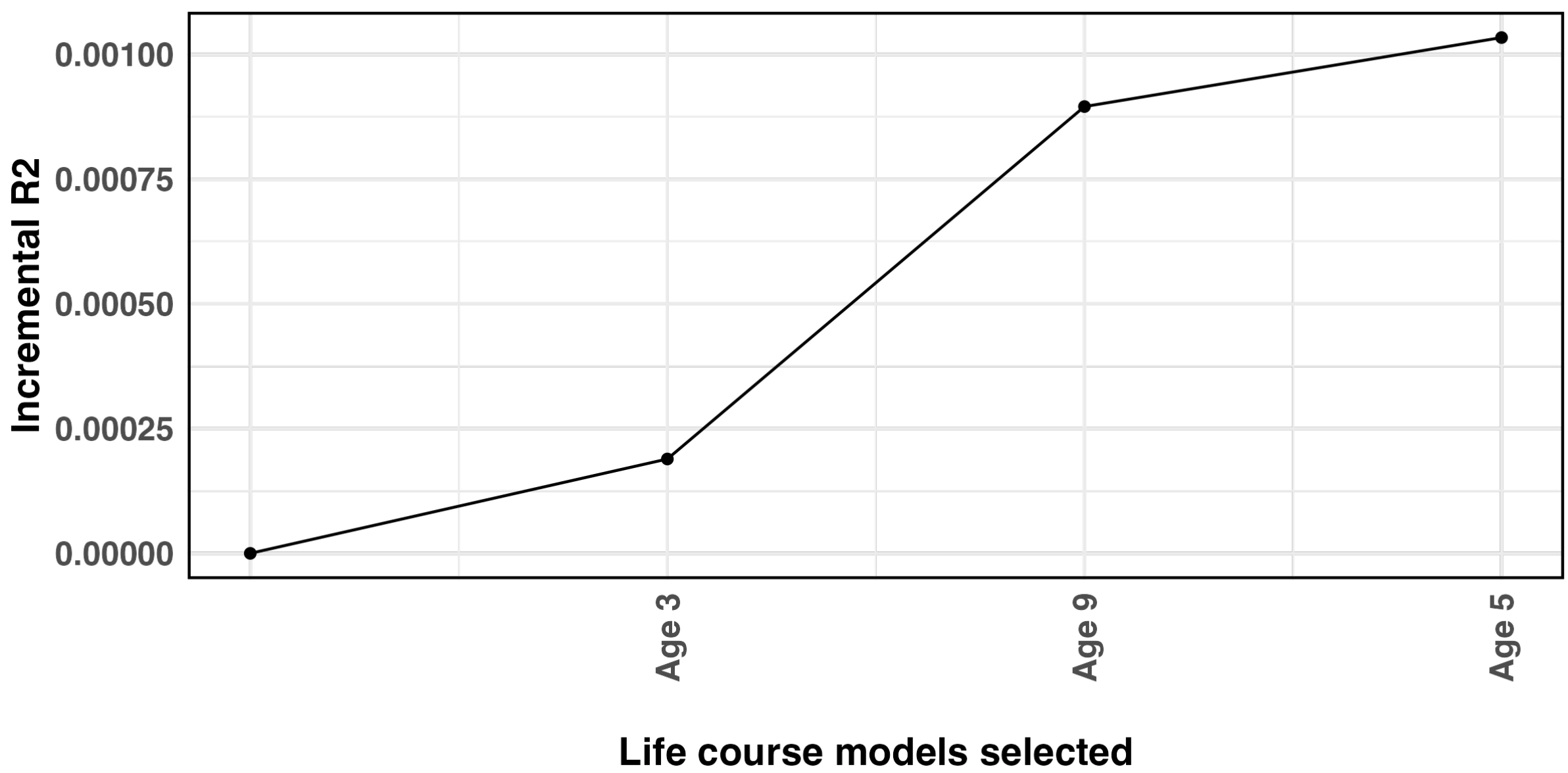

Figure S21: Elbow plot for model selection (Maltreatment)
Sensitivity Analysis: *Cumulative Risk score* summed across three timepoints, with high-adversity categories combined.

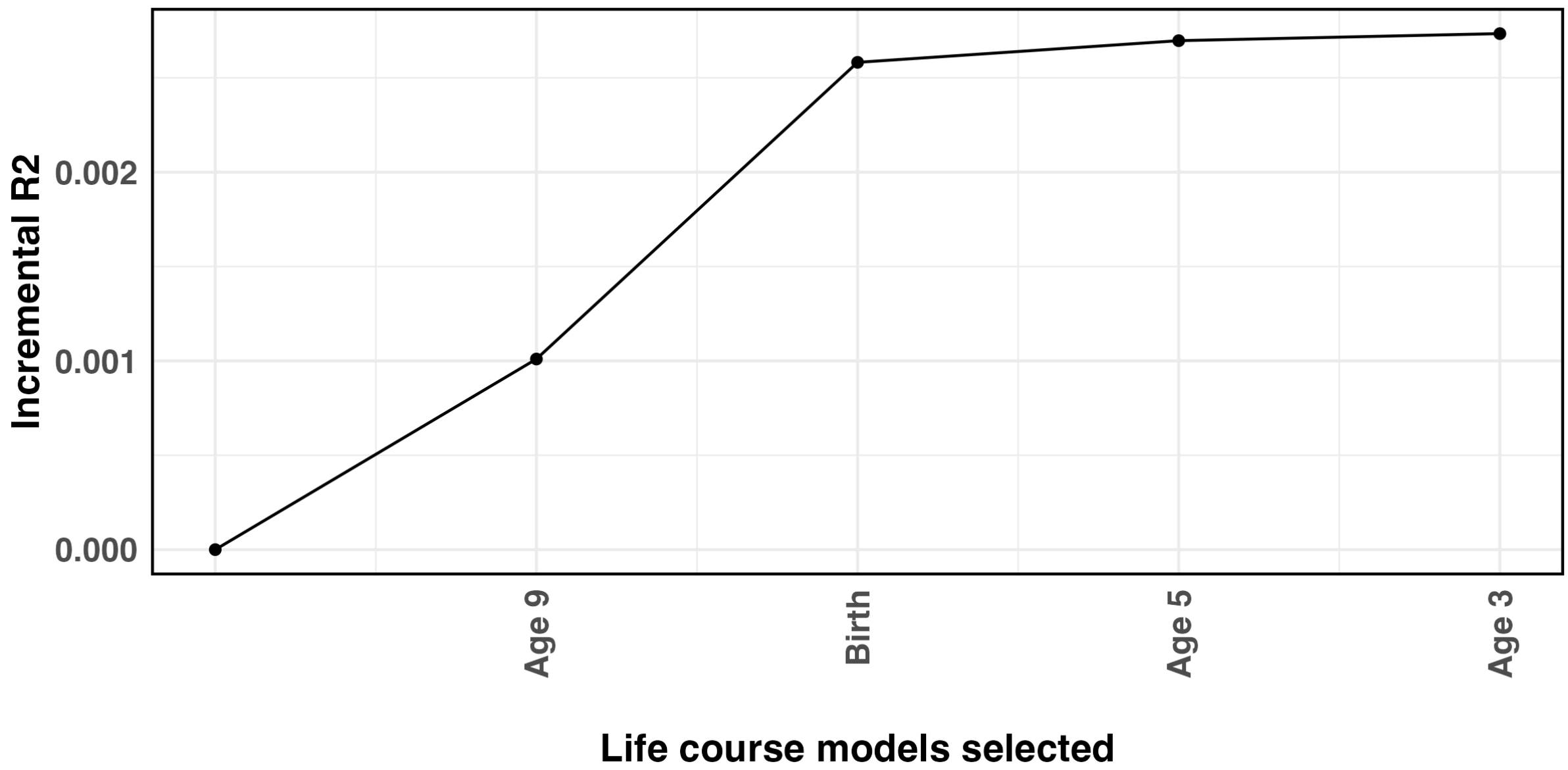

Figure S22: Elbow plot for model selection (Poverty)
Sensitivity Analysis: *Poverty at birth* included within life course model selection procedure.

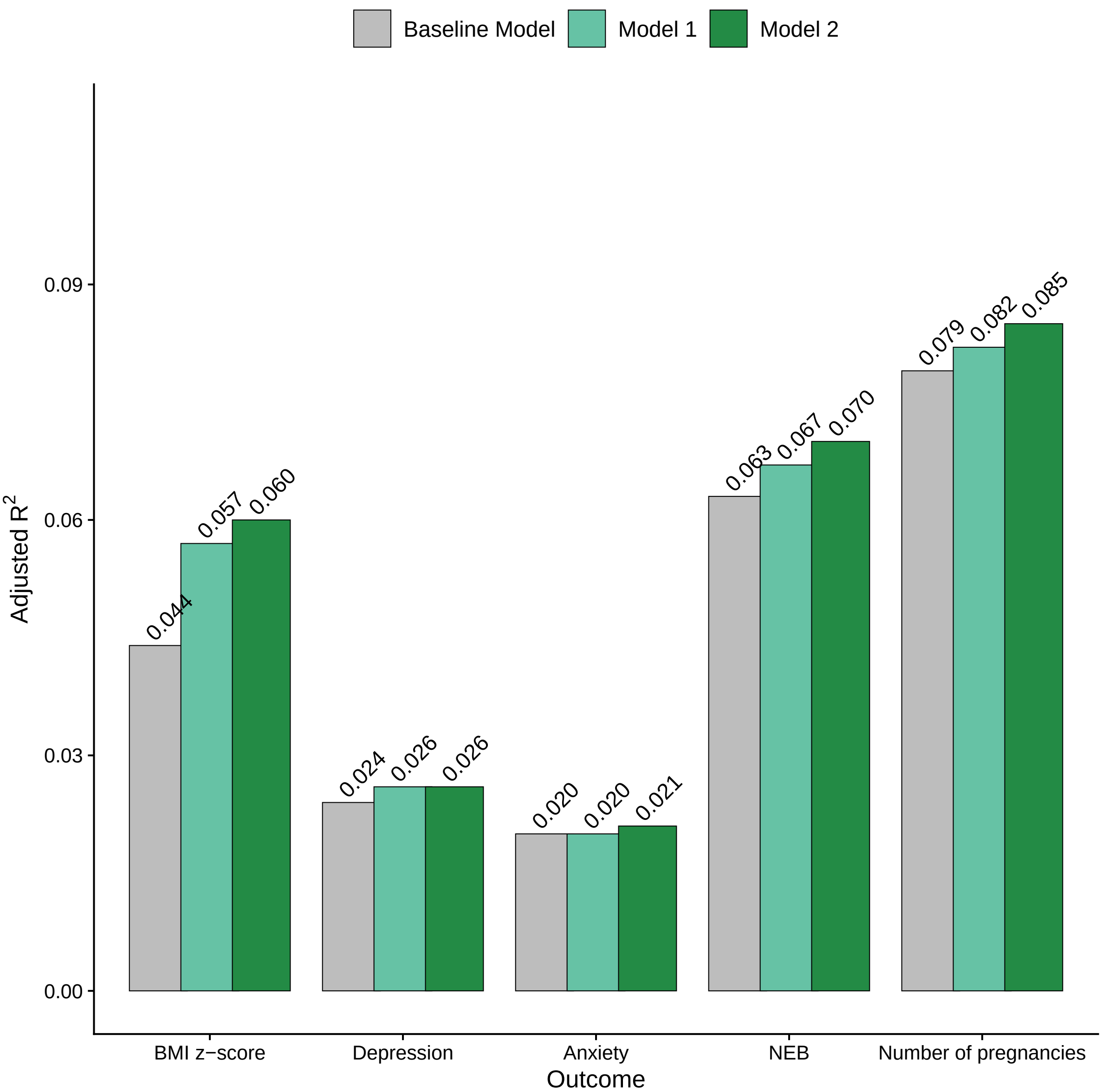

Notes: Bars show adjusted R^2 for the baseline model and models including additional predictors. Incremental increases reflect improvement in model fit when adding the DunedinPACE clock (Model 1) and sensitive ages 3 and 9 (Model 2) to the baseline covariates (sex, age, education, household income, parental income, and race).

Figure S23: Cross-model comparisons of incremental adjusted R^2 by outcomes
